# The Clinical Value of Chemotherapy Combined With Capecitabine in Triple-Negative Breast Cancer—A Meta-Analysis

**DOI:** 10.3389/fphar.2021.771839

**Published:** 2021-11-15

**Authors:** Zilin Zhang, Kai Ma, Jing Li, Yeneng Guan, Chaobo Yang, Aqin Yan, Hongda Zhu

**Affiliations:** ^1^ Key Laboratory of Fermentation Engineering (Ministry of Education), Hubei Key Laboratory of Industrial Microbiology, National “111” Center for Cellular Regulation and Molecular Pharmaceutics, School of Food and Biological Engineering, Hubei University of Technology, Wuhan, China; ^2^ Pharmaceutical Department, Hubei Cancer Hospital, Tongji Medical College, Huazhong University of Science and Technology, Wuhan, China

**Keywords:** chemotherapy, triple-negative breast cancer, capecitabine, meta-analysis, safety

## Abstract

**Purpose:** Triple-negative breast cancer (TNBC) is the most dangerous subtype of breast cancer with high rates of metastasis and recurrence. The efficacy of capecitabine in chemotherapy for TNBC is still controversial. This study evaluated the efficacy and safety of capecitabine combining with standard, adjuvant or neoadjuvant chemotherapy for TNBC.

**Methods:** We systematically searched clinical studies through PubMed, Cochrane library, Embase, Wanfang Database, China Academic Journals (CNKI), and American Society of Clinical Oncology’s (ASCO) annual conference report. Studies were assessed for design and quality by the Cochrane risk of bias tool. A meta-analysis was performed using Review Manager to quantify the effect of capecitabine combined with standard, adjuvant or neoadjuvant chemotherapy on the disease-free survival (DFS) rate and overall survival (OS) rate of TNBC patients. Furthermore, safety analysis was performed to evaluate the adverse events.

**Results:** Twelve randomized controlled clinical trials involving totally 4854 TNBC patients were included, of which 2,214 patients received chemotherapy as control group, and 2,278 patients received capecitabine combining with chemotherapy. The results indicated that capecitabine could significantly improve the DFS [hazard ratio (HR) 0.80, 95% confidence interval (CI) 0.71–0.90, *P* = 0.0003] and OS (HR 0.83, 95% CI 0.74–0.93, *P* = 0.001). In subgroup analysis, the combination of capecitabine and cyclophosphamide exhibited a significant benefit in all outcomes (DFS HR 0.75, 95% CI 0.63–0.90, *P* = 0.002; OS HR 0.65, 95% CI 0.52–0.80, *p <* 0.0001). Additionally, defferent dose of capecitabine subgroup showed same significant effect on the results. Safety analysis showed that the addition of capecitabine was associated with a much higher risk of hand-foot syndrome, diarrhea and mucositis or stomatitis.

**Conclusion:** The results showed that adjuvant capecitabine could bring significant benefits on DFS and OS to unselected TNBC patients, the combination of capecitabine and cyclophosphamide could improve the survival rate of patients, although the addition of capecitabine could bring significant side effects such as hand foot syndrome (HFS) and diarrhea.

## Introduction

Triple-negative breast cancer (TNBC) (10–20% of breast cancer) is a subtype of breast cancer with high rates of metastasis and recurrence and lacks of expression of estrogen receptor (ER), progesterone receptor (PR) and human epidermal growth factor receptor 2 (HER2), which cannot be treated with traditional hormone therapy and Her2-targeted therapy (Li et al., 2018) (Mouh et al., 2016). According to the NCCN (National Comprehensive Cancer Network) guidelines, standard therapeutic strategy for TNBC includes a combination of chemotherapy, surgery, and radiation therapy based on the clinic-pathological features of the disease (Waks and Winer, 2019). Although immunotherapies such as programmed cell death 1 (PD1), programmed cell death ligand 1 (PD-L1) inhibitor have been shown to be effective in the neoadjuvant phase, chemotherapy is the major approved treatment strategy of TNBC (Lebert et al., 2018; Wu et al., 2021). The standard chemotherapy, adjuvant or neoadjuvant chemotherapy methods for TNBC include anthracyclines, taxanes, doxorubicin, and cyclophosphamide, platinum compounds (Lebert et al., 2018; Li et al., 2018), but even with these recognized effective treatments, the risk of relapse of TNBC in 10-years is still up to 20–40% (Howard and Olopade, 2021). Therefore, it is important to explore new adjuvant and neoadjuvant treatment.

Capecitabine is an oral prodrug of fluorouracil, which is converted into the active substance 5-fluorouracil (5-FU) by the higher level of thymidine phosphorylase (TP) in the tumor, it may provide better efficacy and safety due to non-cytotoxic of capecitabine and its intermediates (Ishitsuka et al., 1999). Capecitabine has been approved for the treatment of colorectal cancer, gastric cancer and breast cancer so far (Walko and Lindley, 2005; Iqbal and Pan, 2016). Although capecitabine is still controversial in the treatment of breast cancer, it is one of the widely treatment drug in TNBC neoadjuvant and postoperative adjuvant therapy (Steger et al., 2014; Zhang et al., 2017). Twelve meta-analyses summarized the function of capecitabine in the treatment of breast cancer, most of which included all subtypes of breast cancer. Some analyses showed that capecitabine had no significant effect on breast cancer (Martin et al., 2015; Muss et al., 2019; Lluch et al., 2020), and some randomized controlled trials (RCTs) showed that the addition of capecitabine to chemotherapy could improve the survival rate (Zhang et al., 2015; Joensuu et al., 2017; Masuda et al., 2017; Zhang et al., 2017; Li J. et al., 2020; Wang et al., 2021). At the same time, some analyses showed that the addition of capecitabine couldn’t affect DFS but improve OS (Natori et al., 2017). Two meta-analyses focused on the role of capecitabine in the treatment of TNBC, the results confirmed that the addition of capecitabine could improve DFS and OS in TNBC patients (Li Y. et al., 2020; Huo et al., 2021). However, these meta-analyses were short of the latest updates of relevant clinical trials, and did not show further subgroup analysis such as the effect of capecitabine dose or combination with other chemotherapeutic drugs. It is necessary to enlarge the sample size and refine the subgroup analysis to make the conclusion more robust.

This study evaluated the efficacy and safety of the addition of capecitabine with standard chemotherapy, adjuvant or neoadjuvant chemotherapy for TNBC treatment through meta-analysis, so as to determine whether it could improve the clinical efficacy and reduce adverse reactions. Furthermore, subgroup analysis was conducted to explore the potential benefits of combined cyclophosphamide and capecitabine dose on the clinical efficacy of capecitabine.

## Methods

### Search Criteria

Using “breast cancer” or “triple-negative breast cancer” and “capecitabine” or “Xeloda” as the terms, we searched online databases from inception to October 2021 including PubMed, CNKI, Embase, Wanfang Database and the Cochrane library. The annual conference presentations from American Society of Clinical Oncology (ASCO) were also searched. No language restrictions. The specific search strategy for each database was presented in Supplementary Material S1.

### Inclusion and Exclusion Criteria

#### Type of Studying

Phase II and Phase III clinical randomized controlled trials (RCTs) were included. Observational studies were excluded. These RCTs reported the hazard ratio (HR) and its 95% confidence interval (CI) for DFS and/or OS.

#### Type of Participant

The research subjects were patients with breast cancer (including the TNBC subgroup) or TNBC patients. Eligible patients were females ≥18 years old and confirmed to be TNBC by pathology. There were not any restrictions on other factors of the participants.

#### Type of Interventions

One arm received standard, adjuvant or neoadjuvant chemotherapy, and the other arm received capecitabine in addition to standard, adjuvant or neoadjuvant chemotherapy. Standard chemotherapy or adjuvant or neoadjuvant chemotherapy is defined as chemotherapy with cyclophosphamide, methotrexate, anthracycline, platinum, or taxanes. There were no restrictions on the type, order and dosage of chemotherapy drugs and capecitabine.

#### Type of Comparisons

Based on the definitions of standard, adjuvant and neoadjuvant chemotherapy, capecitabine group and capecitabine-free group were compared in data analysis.

#### Type of Outcome Measures

Primary result: DFS and/or OS and its 95% CIs.

 Adverse events: Any adverse events of any grade.

### Data Collection and Analysis

#### Study Selection

Two researchers independently collected and evaluated all literatures and data. Any disagreement shall be resolved through negotiation or with a third party.

#### Data Extraction

The following data were collected from the included study, including author name, publication time, baseline patient characteristics, treatment plan, DFS, OS and their HRs, 95% CIs, and adverse events. For the same RCT with different authors and different publication years, the most recently published literature data was used. Due to the lack of DFS or OS HR information in some documents, we used Engauge software (version 10.8) and the data processing table provided by Jayne F Tierney to generate survival data based on the survival curve in the report (Tierney et al., 2007).

#### Risk of Bias Assessment

The quality and potential bias of twelve studies was assessed using Cochrane’s bias risk tool. Visualization of results was used by Review Manager software.

#### Statistical Analysis

HR and 95% CIs of the extracted efficacy indicators, and adverse events were incorporated into the meta-analysis. Heterogeneity was assessed using Chi-square test and *I* ^*2*^ test statistics. If *p* < 0.1 or *I* ^2^ > 50%, indicating significant heterogeneity, the random effects model was utilized to merge the studies, otherwise the fixed effects model was used. All trials are two-sided, and the statistical significance is *p* < 0.05. All statistical analysis is performed using Review Manager 5.2 software.

### Subgroup Analysis

The effect of different treatment regimens was compared in subgroup analysis, for example, whether cyclophosphamide was used or not and the effect of the dose of capecitabin in treatment regimen.

### Sensitivity Analysis

Sensitivity analysis was evaluated by re-analyzing after excluded individual studies one by one or changing the statistical model to determine the reliability of the results. The results of sensitivity analysis could be discovered in Supplementary Material S2.

### Publication Bias

Publication bias was analyzed by the Review Manager and presented in the form of a funnel chart.

## Results

### Search Results

**FIGURE 1:**
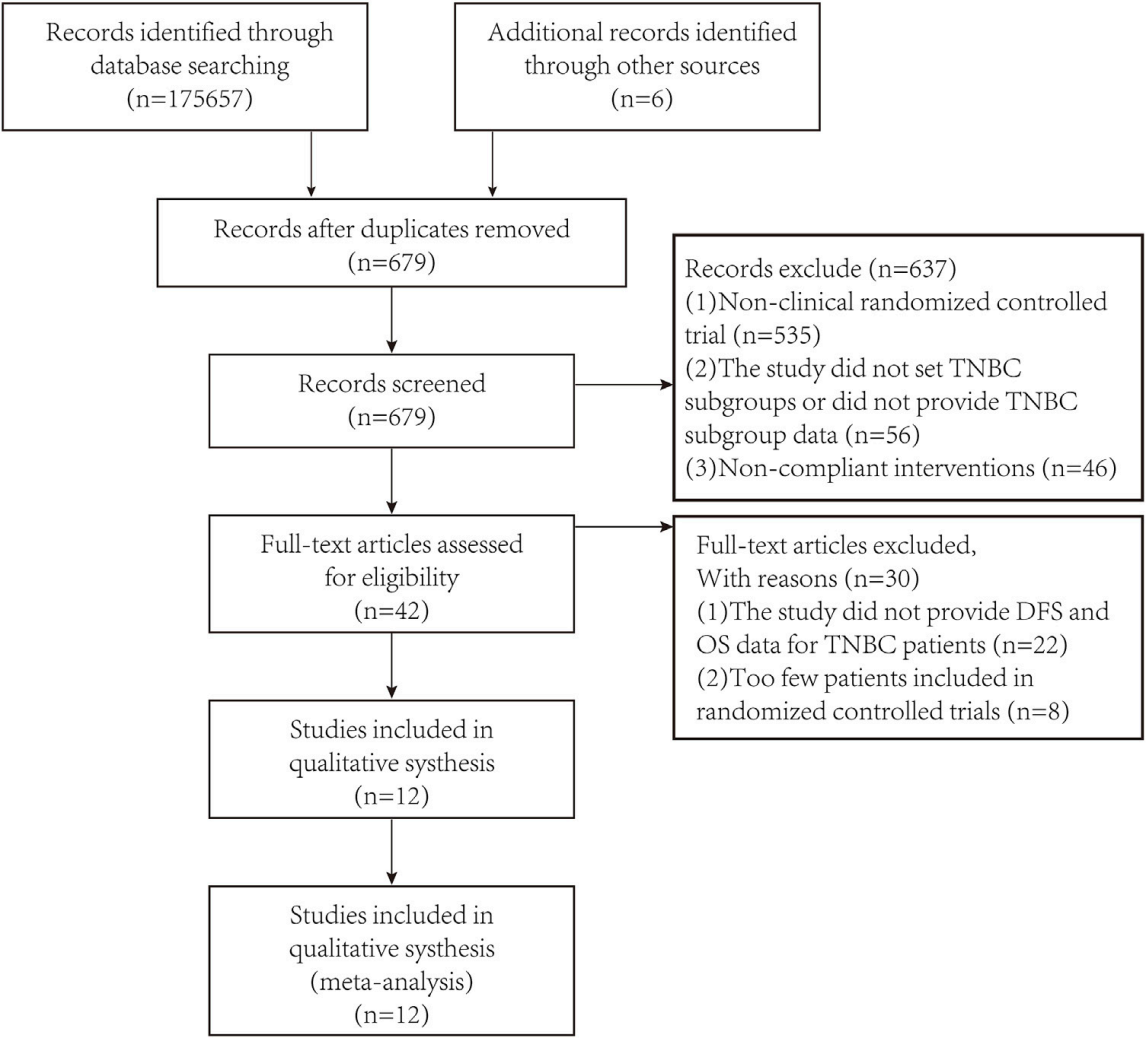
Flow diagram summarizing all study assessment processes.

After preliminary search through the databases and looking at the title and abstract, unqualified studies and repeated studies were excluded based on PRISMA (Preferred Reporting Items for Systematic Reviews and Meta-Analyses). After excluding studies of lower quality and unable to obtain the required data, a total of 12 studies were included in the meta-analysis. The PRISMA flow diagram was shown in Figure 1.

### Characteristics of Included Studies

Twelve relevant RCTs were identified after the initial search. The characteristics were summarized in Table1. The included RCTs comprised of 4 whole cohorts and eight subgroups. A total of 4854 TNBC patients were involved, of which 2,214 patients received standard chemotherapy, adjuvant or neoadjuvant chemotherapy and 2,278 received capecitabine basing on standard chemotherapy, adjuvant or neoadjuvant chemotherapy. The Gepar TRIO trial did not provide a specific number of patients in the TNBC subgroup receiving different treatment modalities (von Minckwitz et al., 2013a). Some experiments only provide OS data or DFS data.

**TABLE 1:** Characteristics of the included studies.

Study	Year	Author	Trial phase	Region	TNBC, N (X/control)	Age	Capecitabine arm	Control arm	Dose of X	Median follow-up (years)	DFS HR/95% CI	OS HR/95% CI	TNBC in study
FinXX Joensuu et al. (2017)	2017	Joensuu Heikki	Ⅲ	America-Europe	93/109	26–65	3TX→3CEX	3T→3CEF	900 mg/m^2^	10.3	0.53 0.31–0.92	0.55 0.31–0.96	Subgroup
GEICAM-2003–10 Martin et al. (2015)	2015	Miguel Martín	Ⅲ	America-Europe	95/71	25–73	4ET→4X	4EC→4T	1,250 mg/m^2^	6.6	1.19 0.70–2.04	NA	Subgroup
CREATE–X Masuda et al. (2017)	2017	N. Masuda	Ⅲ	Asia	139/147	25–74	${\text{standard}}^{1}+6-8\text{X}$	${\text{Standard}}^{1}$	1,250 mg/m^2^	3.6	0.53 0.31–0.92	0.55 0.31–0.96	Subgroup
CBCSG010 Li et al. (2020a)	2020	Junjie Li	Ⅲ	Asia	297/288	18–70	3TX→3CEX	3T→3CEF	1,000 mg/m^2^	5.6	0.66 0.44–0.99	0.67 0.37–1.22	Whole cohort
Zhang et al. Zhang et al. (2015)	2015	Xiaohui Zhang	Ⅱ	Asia	140/140	25–74	4AX	4AC	1,000 mg/m^2^	4.0	1.23 0.41–3.70	0.78 0.20–3.10	Subgroup
USO 01062 O'Shaughnessy et al. (2015)	2015	Joyce O'Sha-ughnessy	Ⅲ	America-Europe	396/384	26–72	4AC→4TX	4AC→4T	825 mg/m^2^	6.4	0.81 0.57–1.15	0.62 0.41–0.94	Subgroup
GEICAM/2003–11_CIBOMA/2004–01 Lluch et al. (2020)	2019	Lluch Ana	Ⅲ	America-Europe	448/428	20–82	8X	None	2,000 mg/m^2^	7.3	0.77 0.59–1.00	0.86 0.63–1.20	Whole cohort
CALGB 49907 Muss et al. (2019)	2019	Muss Hyman B	Ⅲ	America-Europe	76/78	≥65	6X	6CMF/4AC	2,000 mg/m^2^	2.4	NA	0.82 0.53–1.25	Subgroup
SYSUCC-001 Wang et al. (2021)	2020	Xi Wang	Ⅲ	Asia	221/213	24–70	${\text{Standard}}^{2}$ → X	${\text{Standard}}^{2}$	650 mg/m^2^	5.1	0.64 0.42–0.95	0.75 0.47–1.19	Whole cohort
Gepar TRIO von Minckwitz et al. (2013a)	2013	Gunter von Minckwitz	Ⅲ	America-Europe	362	≤36	2TAC→4NX	2TAC→4/6TAC	1,000 mg/m^2^	5.2	0.87 0.61–1.25	NA	Subgroup
GAIN von Minckwitz et al. (2013b)	2013	Gunter von Minckwitz	Ⅲ	America-Europe	213/208	≤65	4EC→4TX	4ETC	2000 mg/m^2^	3.2	0.97 0.68–1.38	0.81 0.54–1.20	Subgroup
ECOG-ACRIN EA1131 Mayer et al. (2021)	2021	Ingrid A. Mayer	Ⅲ	America-Europe	160/148	26–76	6X	4Platinum	1,000 mg/m^2^	1.7	NA	0.98 0.81–1.18	Whole cohort

X capecitabine, T docetaxel, C cyclophosphamide, E epirubicin, F fluorouracil, A pirarubicin, M methotrexate, N vinorelbine.

${\text{Standard}}^{1}$ , Sequential anthracycline and taxane or concurrent anthracycline and taxane or anthracycline-containing chemotherapy only or docetaxel and cyclophosphamide only or fluorouracil plus anthracycline.

${\text{Standard}}^{2}$ , anthracyclines or taxanes based or anthracyclines and taxanes based.

### Risk of Bias Assessment

This meta-analysis was clearly defined through evidence-based medicine methods and PICOS principles. The overall risk of bias for all trials in this study was average. The results of risk of bias were shown in Supplementary Figures S1, S2. Detailed information on the risk of bias assessment was provided in Supplementary Material S3. None of the randomized controlled trials included in this study mentioned clear allocation hiding, blinding of participants and personnel, and blinding of result evaluation, which might affect the results and should be treated with caution.

### Efficacy and Subgroup Analysis

#### DFS

**FIGURE 2:**
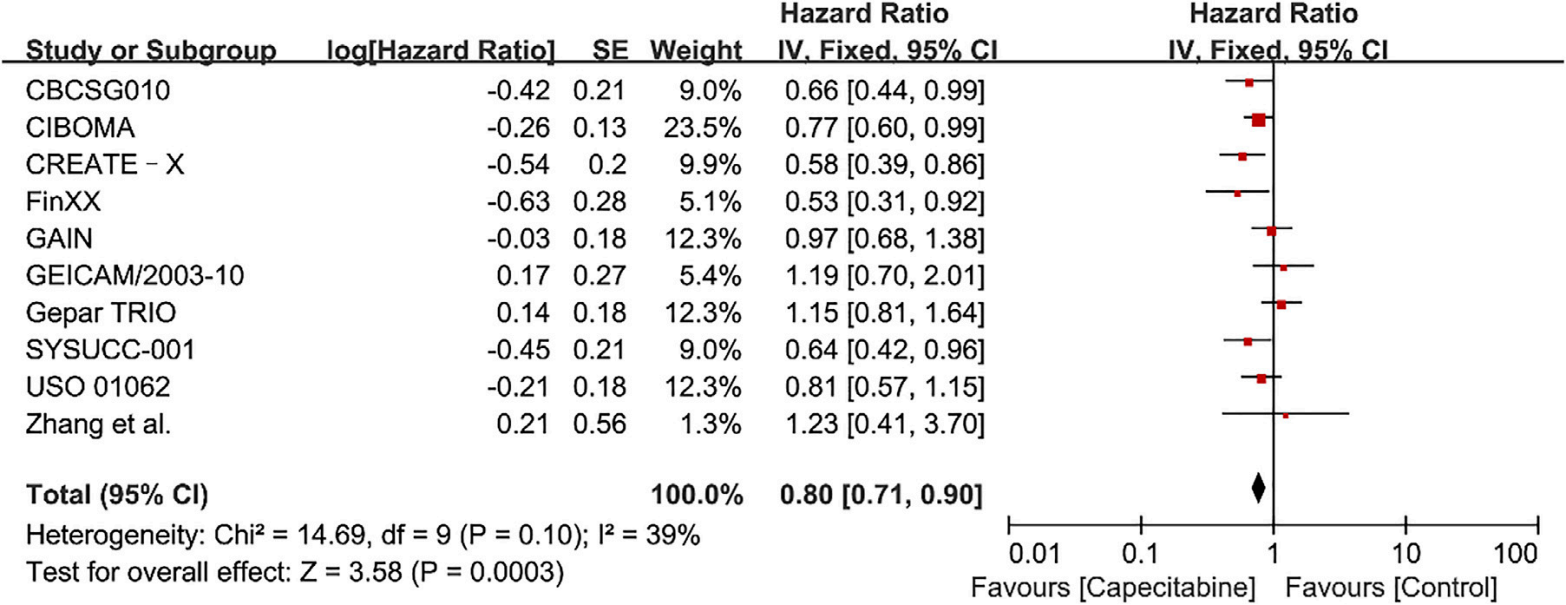
Forest plots for the disease-free survival (DFS) rate in the comparison between chemotherapy with capecitabine group vs. capecitabine-free group in TNBC patients.

The heterogeneity test (Chi^2^ = 14.69, *P* = 0.10, *I* ^2^ = 39%) indicated low statistical heterogeneity between studies. A fixed effects model was applied to calculate the combined HR and 95% CI as 0.80 (0.71–0.90), *P* = 0.0003, indicating a statistically significant difference between capecitabine group and capecitabine-free group (Figure 2). This demonstrated that capecitabine could significantly improve DFS in TNBC patients when combined with chemotherapy, which was consistent with the conclusions of two recent meta-analyses about the role of capecitabine for TNBC treatment (Li Y. et al., 2020; Huo et al., 2021).

**FIGURE 3:**
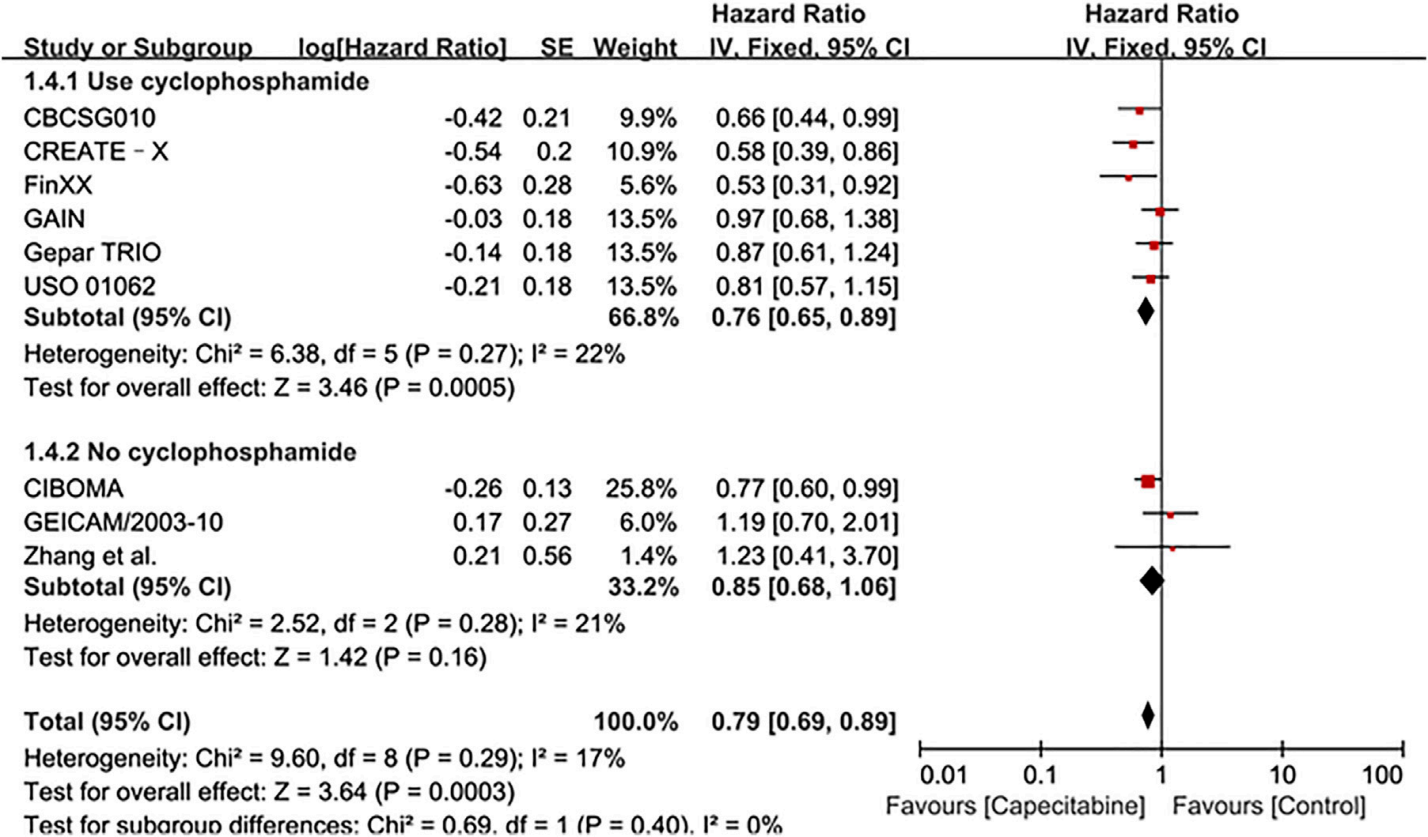
Subgroup analysis of the effect of capecitabine and cyclophosphamide combined with chemotherapy on DFS in TNBC patients.

**FIGURE 4:**
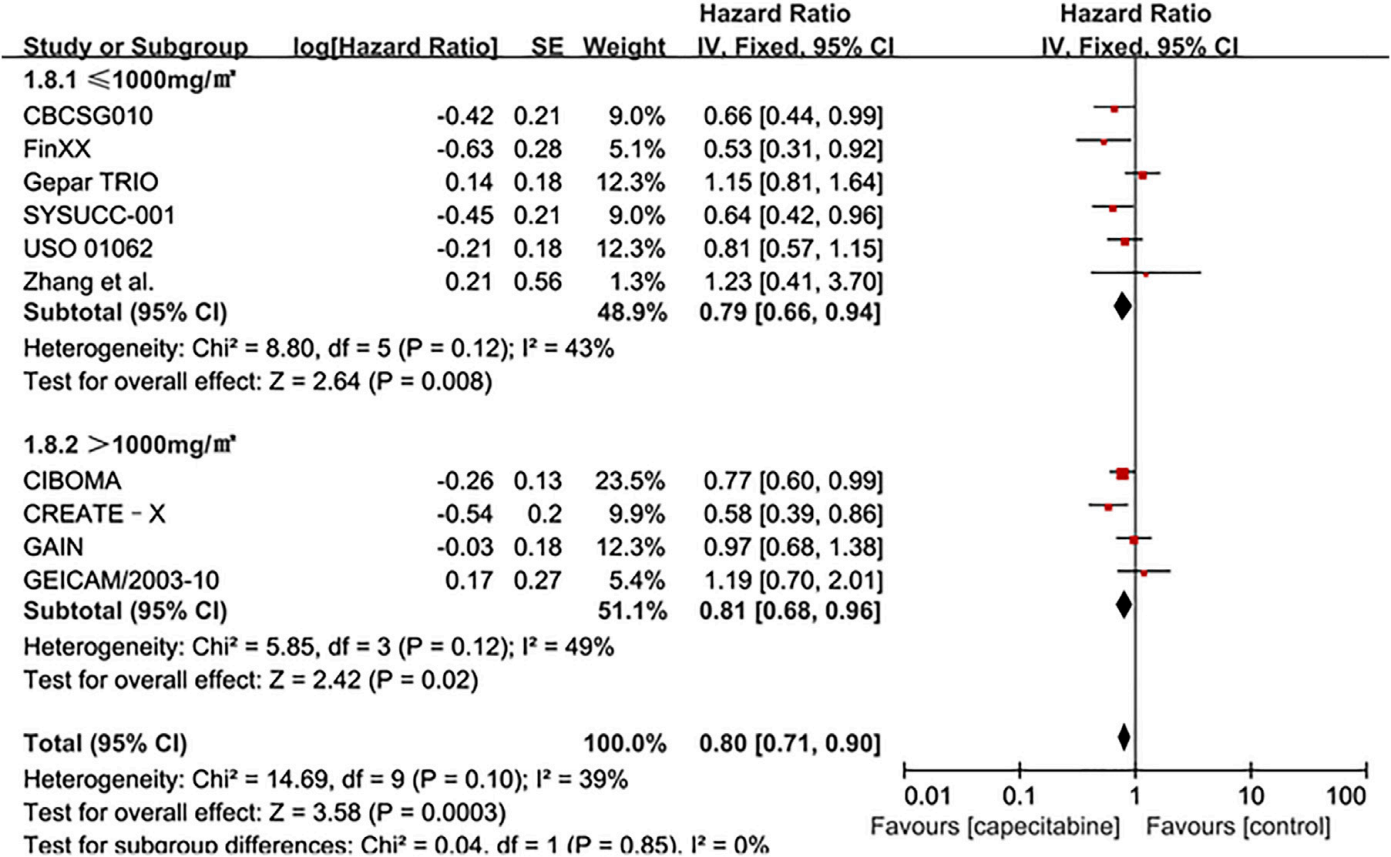
Subgroup analysis of the effect of capecitabine adjuvant chemotherapy dose on DFS in TNBC patients.

Whereas, the addition of capecitabine in the treatment of TNBC still had some negative results and significant side effects (Natori et al., 2017). In order to affirm the potential benefits of capecitabine to TNBC treatment, subgroup analysis was performed based on whether cyclophosphamide was added to adjuvant chemotherapy or the effect of capecitabine dose on adjuvant chemotherapy. Our results showed that a significant improvement in DFS was observed in the combination capecitabine and cyclophosphamide treatment subgroup (HR 0.76, 95% CI 0.65–0.89, *P* = 0.0005), but not in the cyclophosphamide free capecitabine treatment subgroup (HR 0.85, 95% CI 0.68–1.06, *P* = 0.16) (Figure 3). The effect of capecitabine dose on the DFS showed that low dose (<1,000 mg/m^2^) capecitabine had the same significant effect as high dose (>1,000 mg/m^2^) (Figure 4).

#### OS

**FIGURE 5:**
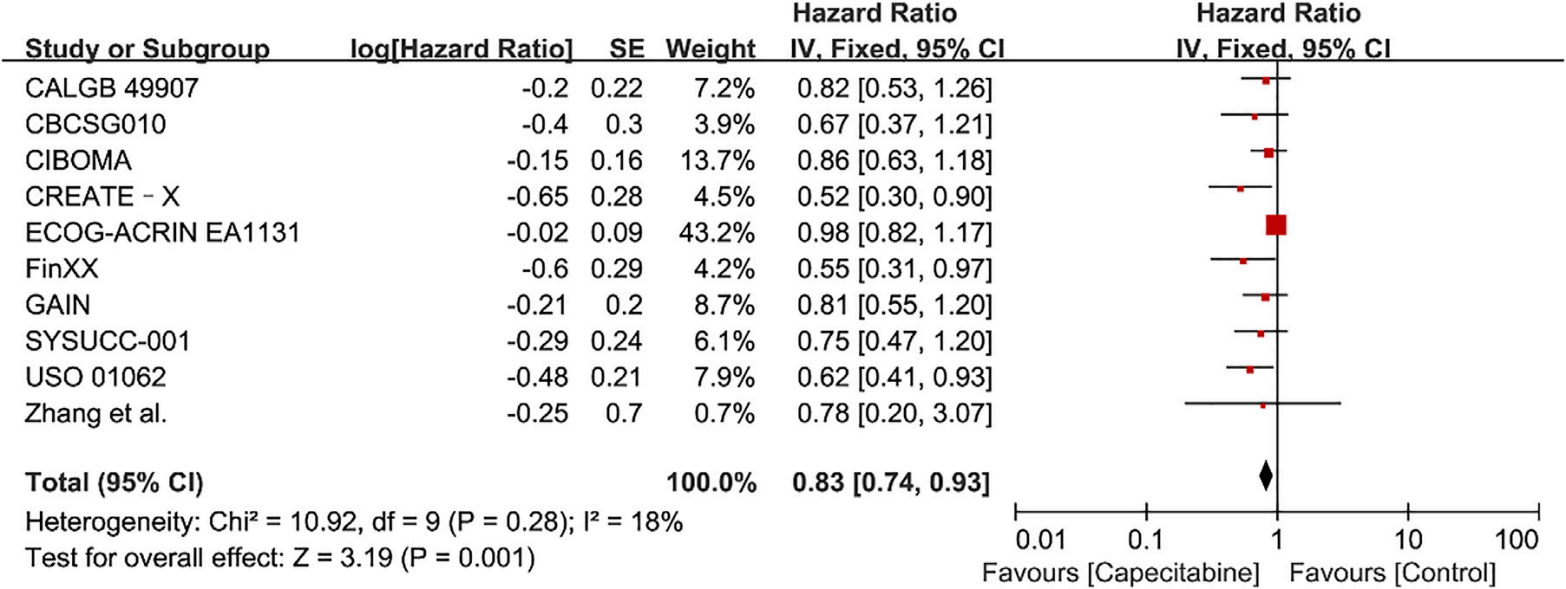
Forest plots for the overall survival (OS) rate in the comparison between chemotherapy with capecitabine group vs. capecitabine-free group in TNBC patients.

**FIGURE 6:**
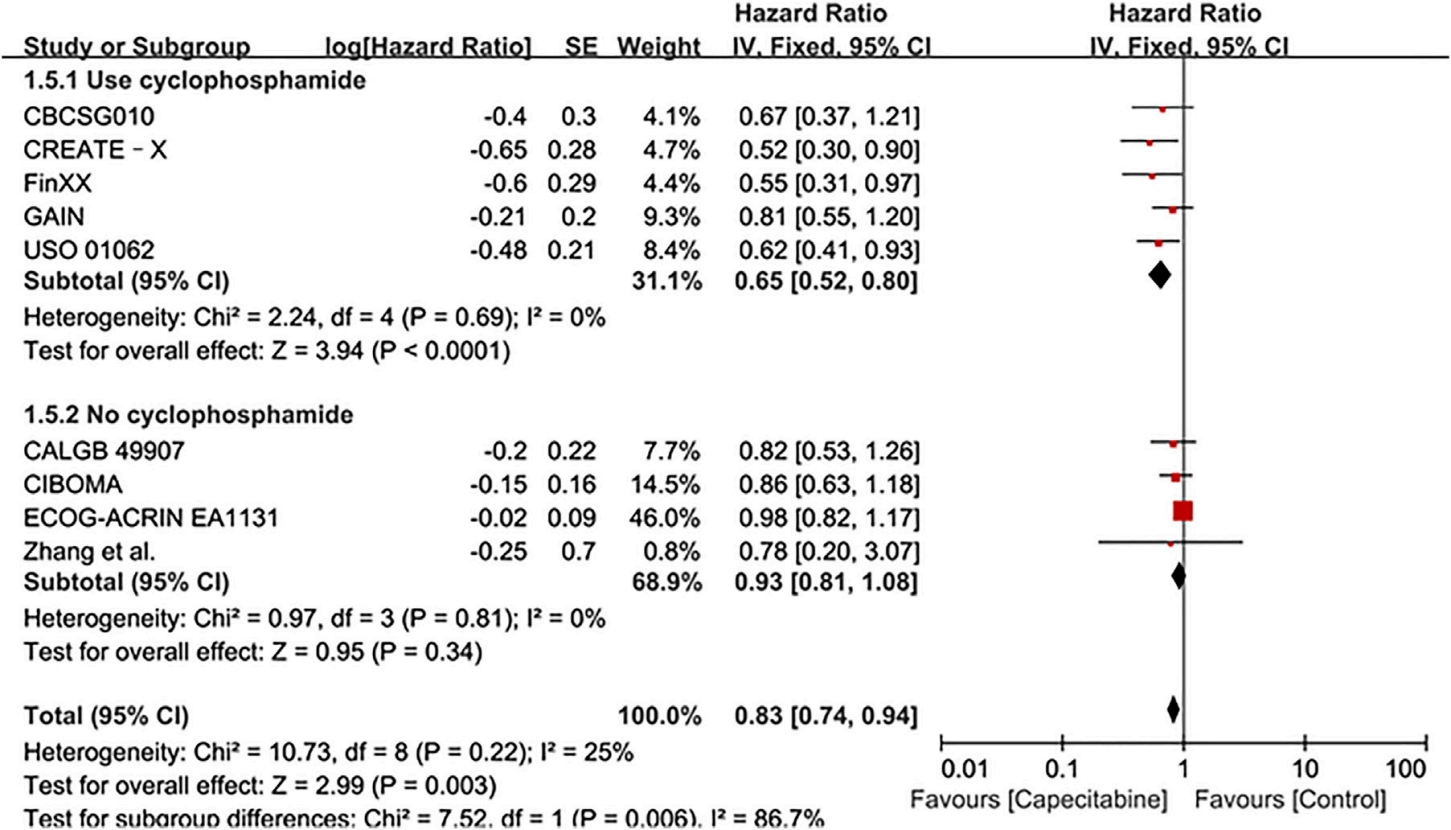
Subgroup analysis of the effect of capecitabine and cyclophosphamide combined with chemotherapy on OS in TNBC patients.

**FIGURE 7:**
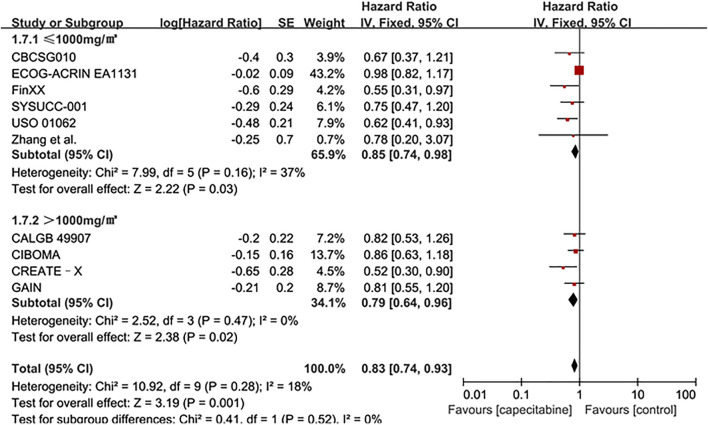
Subgroup analysis of the effect of capecitabine adjuvant chemotherapy dose on OS in TNBC patients.

Ten RCTs were assessed for OS, there was no heterogeneity between the capecitabine group and the capecitabine-free group (Chi^2^ = 10.92, *P* = 0.28, *I* ^2^ = 18%), so a fixed effects model was used to calculate the combined HR and 95% CI as 0.83 (0.74–0.93), *P* = 0.001 (Figure 5). The results suggested that adding capecitabine had a significant improvement in OS. Consistent with the results of DFS subgroup analysis, significant improvement was observed in OS when cyclophosphamide was used (HR 0.65, 95% CI 0.52–0.80, *p* < 0.0001) (Figure 6). Different doses of capecitabine had the same significant improvement in OS (Figure 7).

### Safety and Tolerability

Safety and tolerability analysis of patients with breast cancer included in twelve RCTs was performed. It was found statistically that hand foot syndrome (HFS), neutropenia, mucositis or stomatitis, diarrhea and fatigue were common adverse events with high incidence. Since all adverse reactions between the capecitabine group and the non-capecitabine group were significantly heterogeneous (*p* < 0.05 and *I* ^2^ > 50%), a random effects model was used. The results indicated that capecitabine caused much higher incidence of HFS (OR 25.57, 95% CI 10.44–62.65, *p* < 0.00001), mucositis or stomatitis (OR 1.88, 95% CI 1.06–3.32, *p* = 0.03) and diarrhea (OR 3.66, 95% CI 2.11–6.34, *p <* 0.00001) (Table 2).

**TABLE 2:** Analysis of grade adverse events.

Adverse events	Control n/N	Capecitabine n/N	Odds ratio (95% CI)	*p*
Hand-foot syndrome	82/4,407	834/4,473	25.57 (10.44–62.65)	<0.00001
Neutropenia	1,508/3,769	1,471/3,816	0.85 (0.58–1.24)	0.40
Mucositis or stomatitis	116/3,842	203/3,897	1.88 (1.06–3.32)	0.03
Diarrhea	96/4,407	293/4,473	3.66 (2.11–6.34)	<0.00001
Fatigue	308/4,267	318/4,333	1.02 (0.77–1.35)	0.88

## Discussion

### The Results of Meta-Analysis

The evaluation of the efficacy of capecitabine in breast cancer chemotherapy, including TNBC, has attracted wide attention. For example, the efficacy of two adjuvant chemotherapy regimens, TX + CEX (docetaxel plus capecitabine, cyclophosphamide, epirubicin, and capecitabine) and T + CEF (docetaxel, cyclophosphamide, epirubicin, and fluorouracil), were compared in the FinXX and CBCSG010 trials. The results showed the priority of TX + CEX regimen in DFS (Joensuu et al., 2017; Li J. et al., 2020). Similarly, positive results of DFS and OS were also observed with capecitabine for TNBC patients in the CREATE-X and the USO 01062 trials (O'Shaughnessy et al., 2015; Masuda et al., 2017). The reason might be that nonbasal phenotype tumors with lower value-added index were more sensitive to capecitabine (Lluch et al., 2020). However, for undifferentiated triple-negative patients, the capecitabine group had no improvement in DFS and OS compared with the observation group in the CIBOMA trial. The GEICAM/2003-10 trial showed that capecitabine-free group had the superiority for DFS in lymph node-positive patients (Martin et al., 2015). Similarly, the addition of capecitabine reduced the benefit of lymph node-positive patients in the subgroup analysis of the CIBOMA trial. On the contrary, the different results were obtained in the CBCSG010 trial (Li J. et al., 2020; Lluch et al., 2020). The reason for the different results might be the dose reduction caused by the ethnic difference or the higher risk of recurrence in Asians (Li J. et al., 2020; Lluch et al., 2020), which was similar to the results of the meta-analysis by Li Y et al. (2020). In order to evaluate the efficacy and safety of capecitabine combined with standard chemotherapy, adjuvant chemotherapy or neoadjuvant chemotherapy in the treatment of TNBC, it is necessary to enlarge the sample size and refine the subgroup analysis, so as to make the conclusion more reliable.

Herein, a meta-analysis was performed to evaluate the potential benefits of the clinical efficacy of capecitabine for TNBC. Twelve RCTs were retrieved and included for analysis according to evidence-based medicine methods and PICOS principles. The research was evaluated by bias risk assessment and the overall level of the included studies was average. The results showed that adjuvant capecitabine could bring significant benefits on DFS and OS to unselected TNBC patients, the combination of capecitabine and cyclophosphamide could improve the survival rate of patients, although the addition of capecitabine could bring significant side effects such as HFS and diarrhea. Taxanes and cyclophosphamide as first-line drugs for breast cancer chemotherapy can up-regulate the activity of thymidine phosphorylase (TP) in the tumor (Kurosumi et al., 2000). Cyclophosphamide in standard, adjuvant or neoadjuvant chemotherapy regimens including capecitabine may up-regulate ThdPase to promote the conversion of capecitabine to fluorouracil and improve the efficacy of capecitabine (Khodeer et al., 2020; Refaie et al., 2020).

The dose of capecitabine or the duration of capecitabine treatment, and even the discontinuation of capecitabine due to early toxicity is one of the influencing factors. The SYSUCC-001 trial showed that adding low-dose capecitabine as maintenance therapy after standard adjuvant therapy significantly improved disease-free survival (Wang et al., 2021). However, two randomized controlled trials were designed with the same dose of capecitabine (1000 mg/m^2^, twice a day), the former had six cycles and the latter had eight cycles. Although the proportion of patients in the capecitabine group who reduced the dose was similar in the two trials (39.1 vs. 36.9%), the former reduced the dose less and the proportion of patients who completed the complete planned cycle was greater (84.9 vs. 75.2%). The results proved that the duration of capecitabine treatment might have a significant impact on the results (Wang et al., 2021). The addition of high-dose capecitabine in the CALGB 49907 elderly breast cancer trial showed negative results. It not only brought a lower survival rate, but also induced more obvious side effects. Most deaths were caused by non-breast cancer, which might be related to other competing death factors caused by age and obvious side effects (Muss et al., 2019). Since there was no more rigorous distinction between baseline characteristics such as age and ethnicity, which might affect the patient’s dose, there might be some deviations in the results. In spite of different dose of capecitabine subgroup analysis showed same significant effect in our analysis (Figures 4, 7), the addition of capecitabine was associated with higher adverse events such as hand-foot syndrome, diarrhea and mucositis or stomatitis (Table 2). Our analysis suggested that low dose (<1,000 mg/m^2^) capecitabine combined with cyclophosphamide was more beneficial for TNBC patients.

Some research reports indicated that specific TNBC subgroups, including specific genes related to anti-tumor immunity, immune response, and capecitabine activation might gain greater improvement from the addition of capecitabine (Asleh et al., 2020). In the ABCSG-24 trial, preoperative use of capecitabine increased pathologic complete response (pCR) rates. For the TNBC subgroup, this improvement was more significant (Steger et al., 2014). For some patients with special baseline characteristics, the benefits of capecitabine may be more obvious according to more clinical data and more rigorous analysis. The positive efficacy of adding capecitabine might depend on patient’s race, age and different clinical characteristics of patients (Zhang et al., 2016; Li Y. et al., 2020; Huo et al., 2021).

Compared to other meta-analysis, we included more data and performed other subgroup analysis including the effect of capecitabine and cyclophosphamide in combination and the influence of capecitabine dose on adjuvant chemotherapy (Zhang et al., 2016; Natori et al., 2017; Li Y. et al., 2020; Huo et al., 2021). The meta-analysis by Yan Li et al. focused on the role of adjuvant capecitabine in standard chemotherapy, the influence of region and treatment period on the effect of capecitabine were analyzed in the subgroup. The results showed that capecitabine improved the survival of TNBC patients regardless of the region. Longer treatment cycle had a significant improvement for DFS but did not affect OS (Li Y. et al., 2020). The meta-analysis by Huo et al. analyzed the effects of capecitabine in adjuvant and neoadjuvant chemotherapy and different lymph node status on the effect of capecitabine (Huo et al., 2021). The results showed that the addition of capecitabine, lymph node positive and adjuvant chemotherapy were beneficial for DFS, which might be related to the anti-angiogenesis of capecitabine and the inhibition of tumor immune escape (Pasquier et al., 2010). The ECOG-ACRIN EA1131 trial compared the effects of platinum preparations and capecitabine after neoadjuvant chemotherapy (Mayer et al., 2021). The results showed that there was no significant difference between the effects of platinum preparations and capecitabine, and platinum preparations brought more serious toxicity.

### Heterogeneity of Research and Publication Bias

**FIGURE 8:**
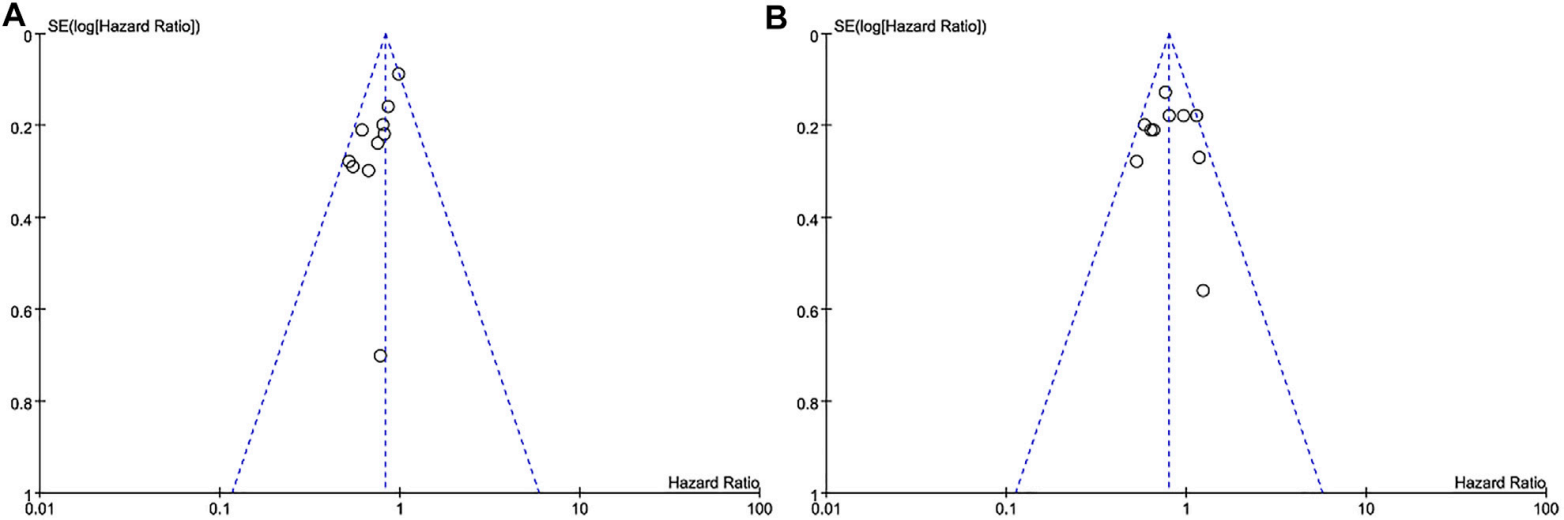
Funnel chart of publication bias. **(A)** DFS. **(B)** OS.

The subtypes of triple-negative breast cancer, the diversity of treatment options, and other baseline characteristics of patients were the main reasons for heterogeneity of the included studies, the results were inevitably. Most of *I* ^*2*^ in our analysis was less than 50%, indicating low heterogeneity of results between studies. The publication bias was displayed in the form of a funnel diagram with small sample size and a certain publication bias (Figure 8).

### Limitation

We have tried our best to ensure the reliability of the results in our research, but there were still some limitations inevitably. Firstly, many randomized controlled trials were not included due to lack of enough data, and the quality of the included studies was average. Secondly, the intervention measures of the randomized controlled trials included in the analysis and the baseline characteristics of patients were inconsistent, which might affect the results. Expand the sample size and refine the subgroup analysis will make the conclusion more reliable.

## Data Availability

The original contributions presented in the study are included in the article/Supplementary Material, further inquiries can be directed to the corresponding authors.
