# Supplementary material for: The Clinical Value of Chemotherapy Combined With Capecitabine in Triple-Negative Breast Cancer—A Meta-Analysis
Source: Front Pharmacol. 2021 Nov 15;12:771839. doi: 10.3389/fphar.2021.771839 (PMC8634095; doi:10.3389/fphar.2021.771839)
Supplement: Supplementary file 1 [file DataSheet1.DOCX]

The clinical value of chemotherapy combined with capecitabine in triple-negative breast cancer—A meta-analysis

Supplementary Material

Zilin Zhang^1^, Kai Ma^1^, Jing L^2^, Yeneng Guan^1^, Chaobo Yang^1^, Aqing Yan^1^, Hongda Zhu^1*^

^1^ Key Laboratory of Fermentation Engineering (Ministry of Education), National “111” Center for Cellular Regulation and Molecular Pharmaceutics, Hubei Key Laboratory of Industrial Microbiology, School of Food and Biological Engineering, Hubei University of Technology, Wuhan, China.

^2^ Pharmaceutical Department, Hubei Cancer Hospital, Tongji Medical College, Huazhong University of Science and Technology, Wuhan, China.

*** Correspondence:** Hongda Zhu
bszzhuhongda@yeah.net

**Supplementary material 1**

The specific search strategy for each database.

1. PubMed

| Supplementary material 1: Search strategies for databases. | | | | |  |  |
| --- | --- | --- | --- | --- | --- | --- |
| Search number | Query | Sort By | Filters | Search Details | Results | Time |
| 1 | ("breast cancer" AND capecitabine) OR ("breast cancer" AND xeloda)) OR ("triple-negative breast cancer" AND capecitabine)) OR ("triple-negative breast cancer" AND xeloda) |  | Abstract, Free full text, Full text | ("breast cancer"[All Fields] AND ("capecitabine"[MeSH Terms] OR "capecitabine"[All Fields] OR "capecitabin"[All Fields])) OR ("breast cancer"[All Fields] AND ("capecitabine"[MeSH Terms] OR "capecitabine"[All Fields] OR "capecitabin"[All Fields] OR "xeloda"[All Fields])) OR ("triple-negative breast cancer"[All Fields] AND ("capecitabine"[MeSH Terms] OR "capecitabine"[All Fields] OR "capecitabin"[All Fields])) OR ("triple-negative breast cancer"[All Fields] AND ("capecitabine"[MeSH Terms] OR "capecitabine"[All Fields] OR "capecitabin"[All Fields] OR "xeloda"[All Fields])) | 1,824 | 7:25:44 |

2. Embase


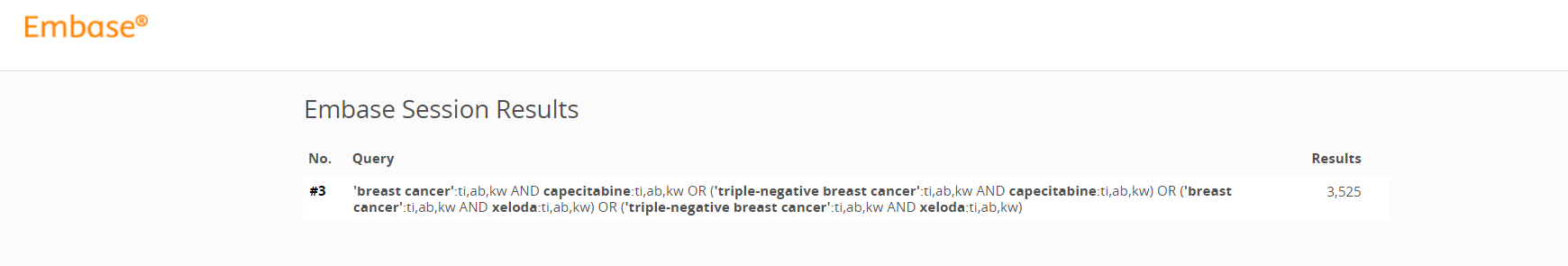


3. Cochrane


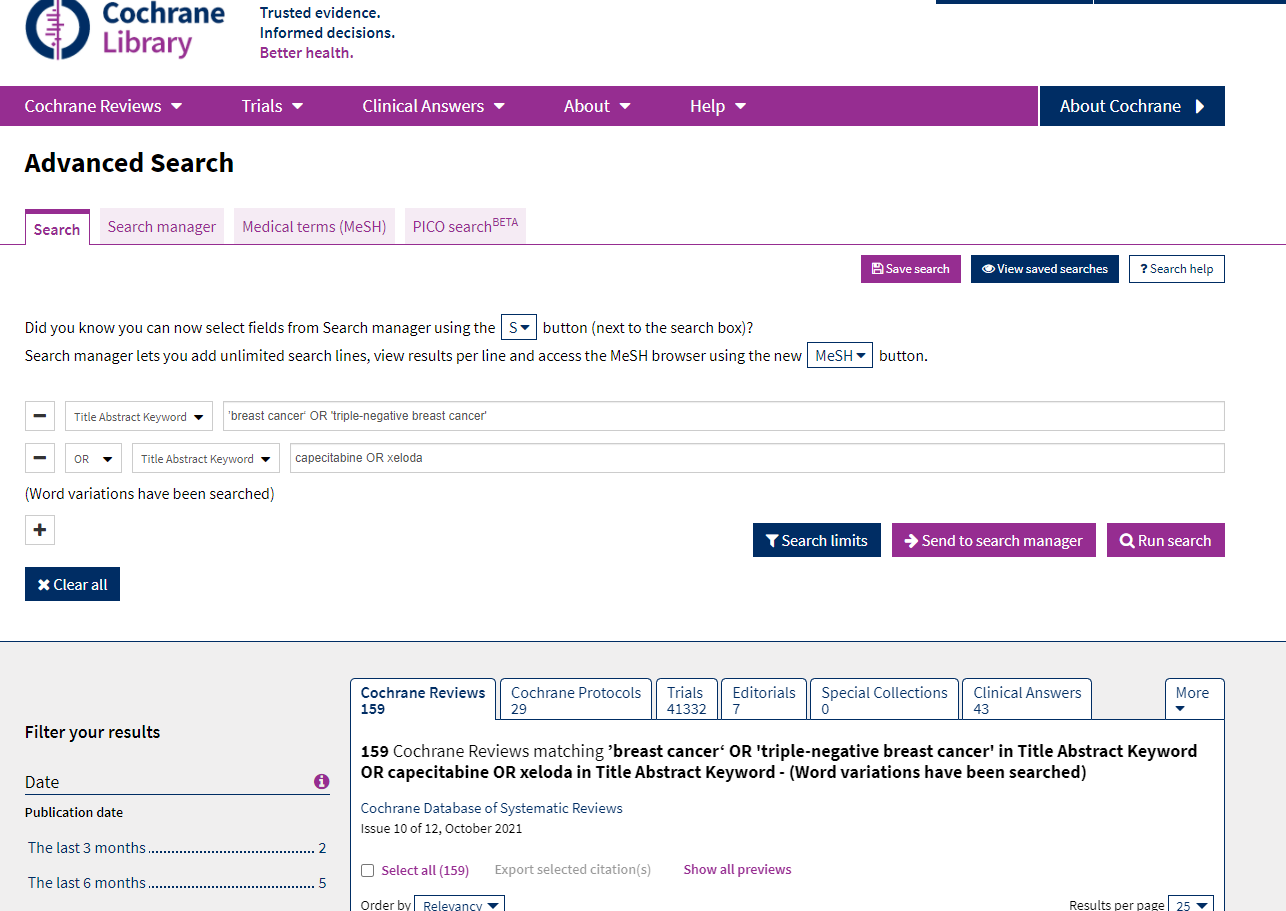


4. ASCO


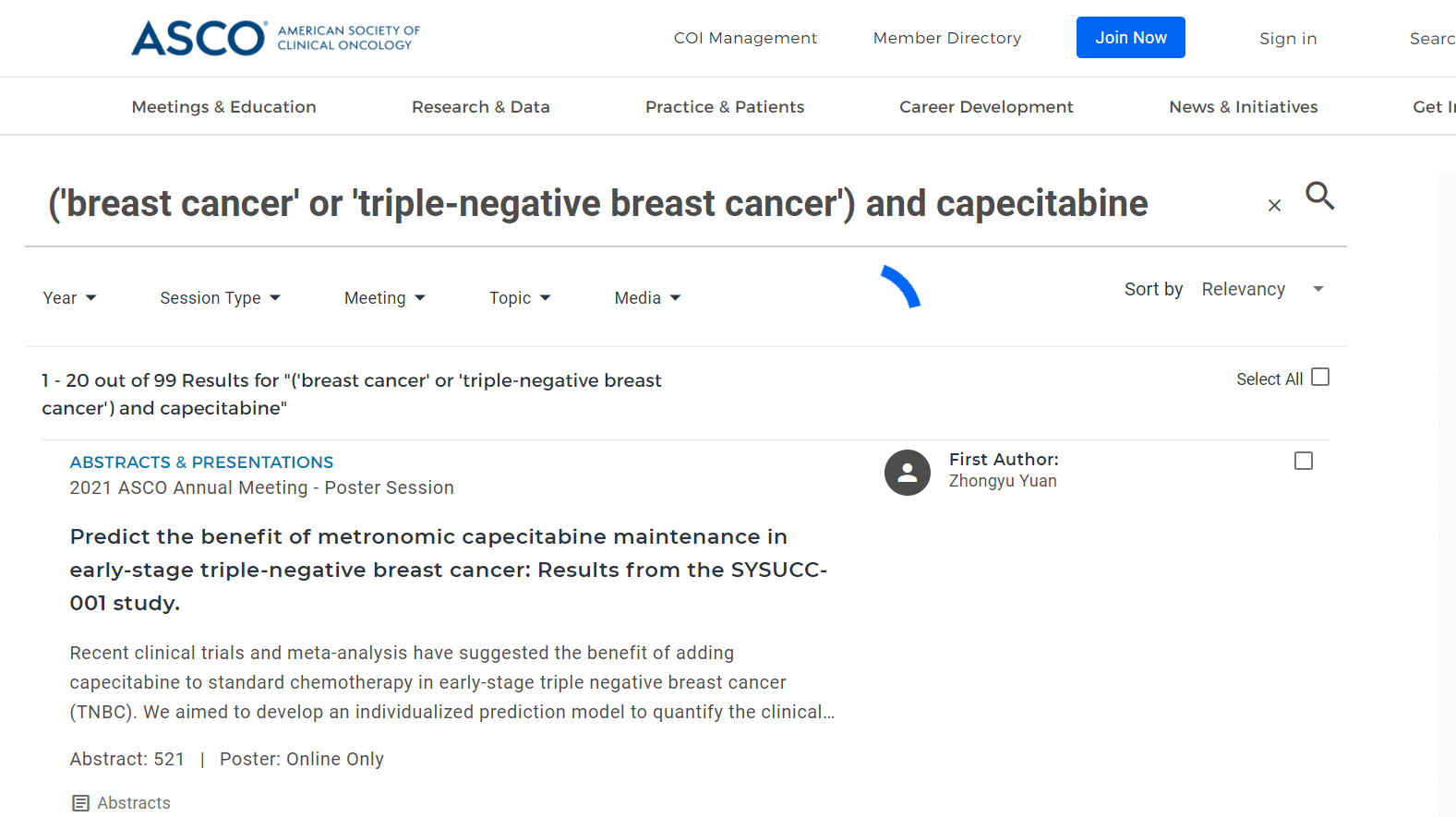


5. CNKI


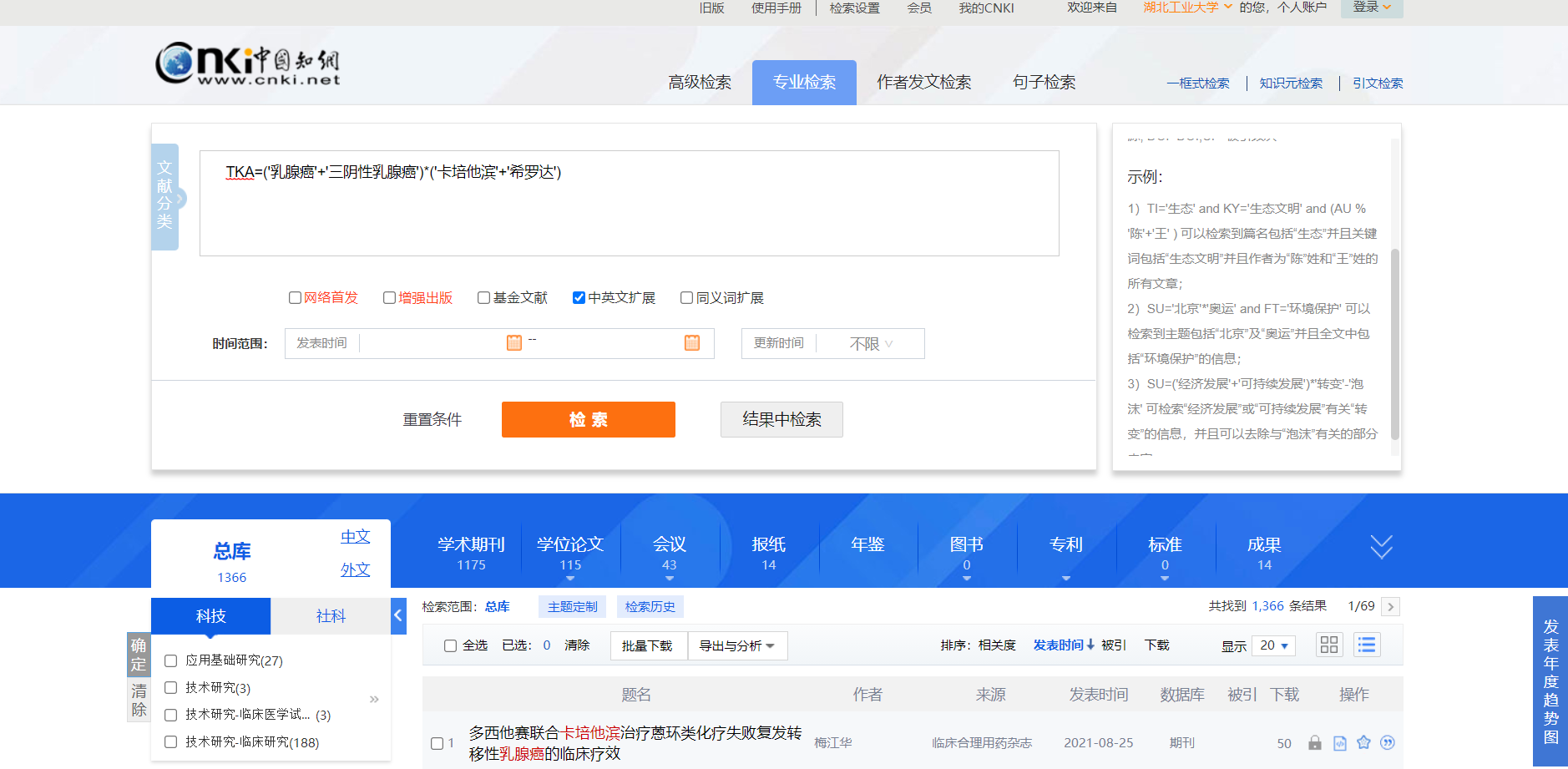


6. Wanfang database


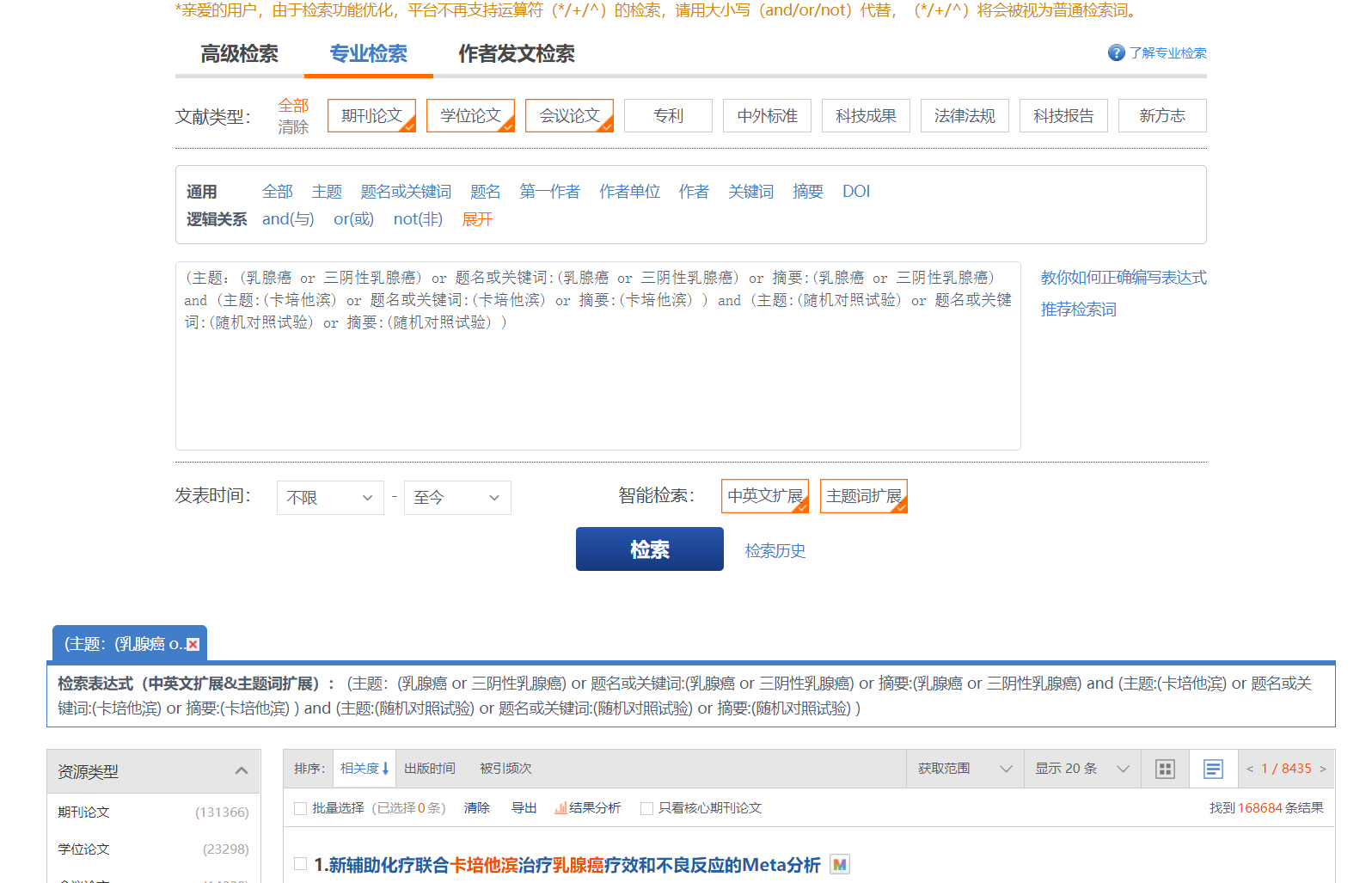


**Supplementary material 2**

The results of sensitivity analysis

DFS

| Analytical method | Study | Statistical model | HR | 95% CI | P value |
| --- | --- | --- | --- | --- | --- |
| Exclude a certain study | FinXX | Fixed effects model | 0.82 | 0.72-0.93 | 0.002 |
|  |  | Random effects model | 0.82 | 0.69-0.97 | 0.02 |
|  | CREATE–X | Fixed effects model | 0.83 | 0.73-0.94 | 0.004 |
|  |  | Random effects model | 0.83 | 0.70-0.98 | 0.03 |
|  | SYSUCC-001 | Fixed effects model | 0.82 | 0.72-0.93 | 0.002 |
|  |  | Random effects model | 0.82 | 0.68-0.97 | 0.02 |
|  | CBCSG010 | Fixed effects model | 0.81 | 0.71-0.93 | 0.002 |
|  |  | Random effects model | 0.81 | 0.68-0.97 | 0.02 |
|  | CIBOMA | Fixed effects model | 0.81 | 0.70-0.93 | 0.003 |
|  |  | Random effects model | 0.80 | 0.66-0.98 | 0.03 |
|  | USO 01062 | Fixed effects model | 0.80 | 0.70-0.91 | 0.0007 |
|  |  | Random effects model | 0.79 | 0.66-0.96 | 0.02 |
|  | GAIN | Fixed effects model | 0.78 | 0.68-0.89 | 0.0002 |
|  |  | Random effects model | 0.78 | 0.65-0.93 | 0.005 |
|  | Gepar TRIO | Fixed effects model | 0.76 | 0.66-0.87 | ＜0.0001 |
|  |  | Random effects model | 0.76 | 0.65-0.88 | 0.0004 |
|  | GEICAM/2003-10 | Fixed effects model | 0.78 | 0.69-0.89 | 0.0001 |
|  |  | Random effects model | 0.77 | 0.66-0.91 | 0.002 |
|  | Zhang et al. | Fixed effects model | 0.79 | 0.70-0.90 | 0.0003 |
|  |  | Random effects model | 0.79 | 0.67-0.94 | 0.006 |
| Change the statistical model |  | Fixed effects model | 0.80 | 0.71-0.90 | 0.0003 |
|  |  | Random effects model | 0.80 | 0.68-0.94 | 0.007 |

OS

| Analytical method | Study | Statistical model | HR | 95% CI | P value |
| --- | --- | --- | --- | --- | --- |
| Exclude a certain study | FinXX | Fixed effects model | 0.84 | 0.75-0.95 | 0.005 |
|  |  | Random effects model | 0.82 | 0.72-0.94 | 0.004 |
|  | CREATE–X | Fixed effects model | 0.85 | 0.75-0.95 | 0.006 |
|  |  | Random effects model | 0.84 | 0.75-0.95 | 0.006 |
|  | SYSUCC-001 | Fixed effects model | 0.83 | 0.74-0.94 | 0.003 |
|  |  | Random effects model | 0.79 | 0.67-0.92 | 0.003 |
|  | CBCSG010 | Fixed effects model | 0.84 | 0.74-0.94 | 0.003 |
|  |  | Random effects model | 0.79 | 0.68-0.92 | 0.003 |
|  | CIBOMA | Fixed effects model | 0.82 | 0.73-0.93 | 0.002 |
|  |  | Random effects model | 0.76 | 0.65-0.90 | 0.002 |
|  | USO 01062 | Fixed effects model | 0.85 | 0.75-0.96 | 0.008 |
|  |  | Random effects model | 0.83 | 0.72-0.95 | 0.006 |
|  | GAIN | Fixed effects model | 0.83 | 0.73-0.94 | 0.003 |
|  |  | Random effects model | 0.78 | 0.66-0.91 | 0.002 |
|  | ECOG-ACRIN EA1131 | Fixed effects model | 0.73 | 0.62-0.85 | ＜0.0001 |
|  |  | Random effects model | 0.73 | 0.62-0.85 | ＜0.0001 |
|  | CALGB 49907 | Fixed effects model | 0.83 | 0.73-0.93 | 0.002 |
|  |  | Random effects model | 0.0.78 | 0.66-0.91 | 0.002 |
|  | Zhang et al. | Fixed effects model | 0.83 | 0.74-0.93 | 0.001 |
|  |  | Random effects model | 0.78 | 0.67-0.91 | 0.001 |
| Change the statistical model |  | Fixed effects model | 0.83 | 0.74-0.93 | 0.001 |
|  |  | Random effects model | 0.79 | 0.69-0.91 | 0.001 |

**Supplementary material 3**

Detailed information on the risk of bias assessment


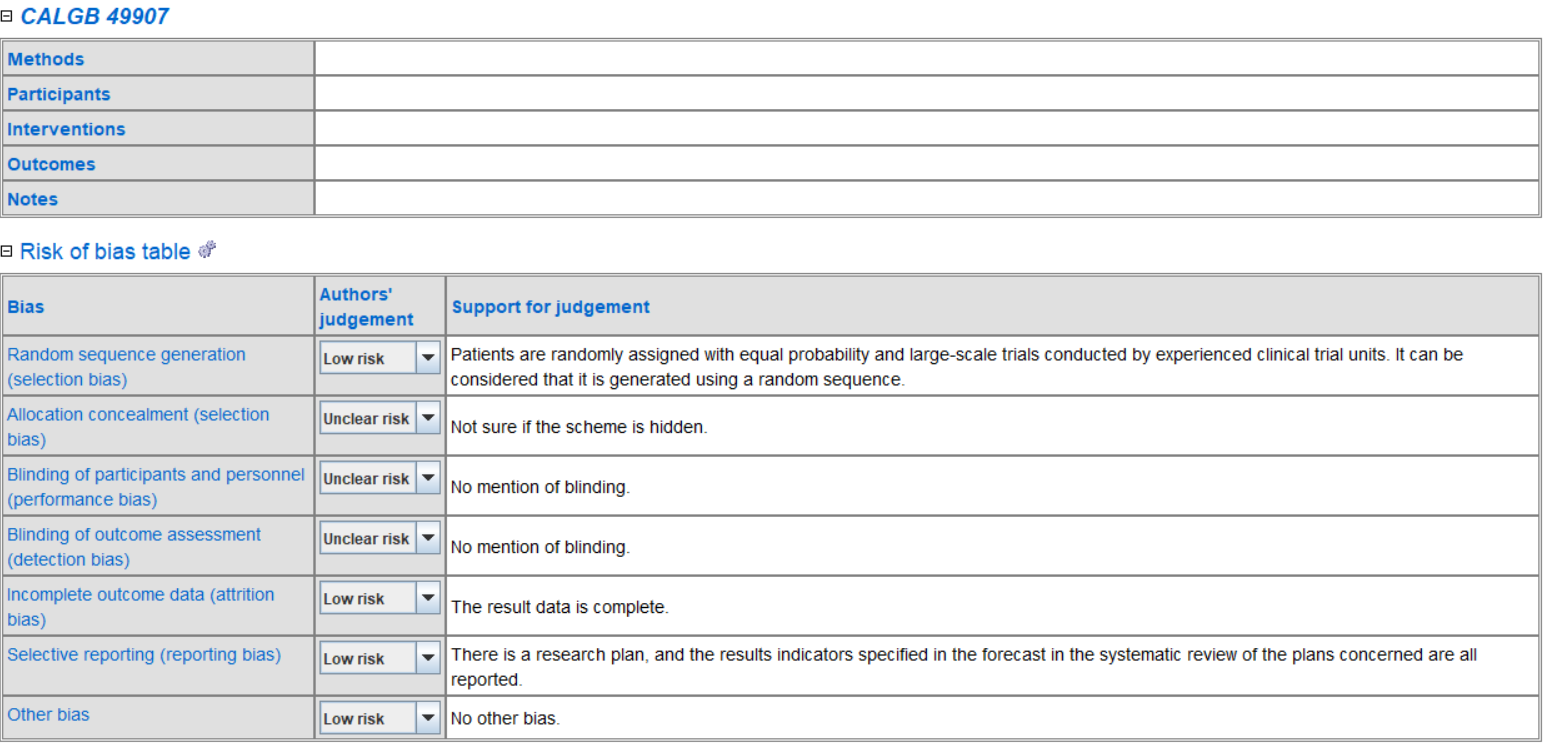

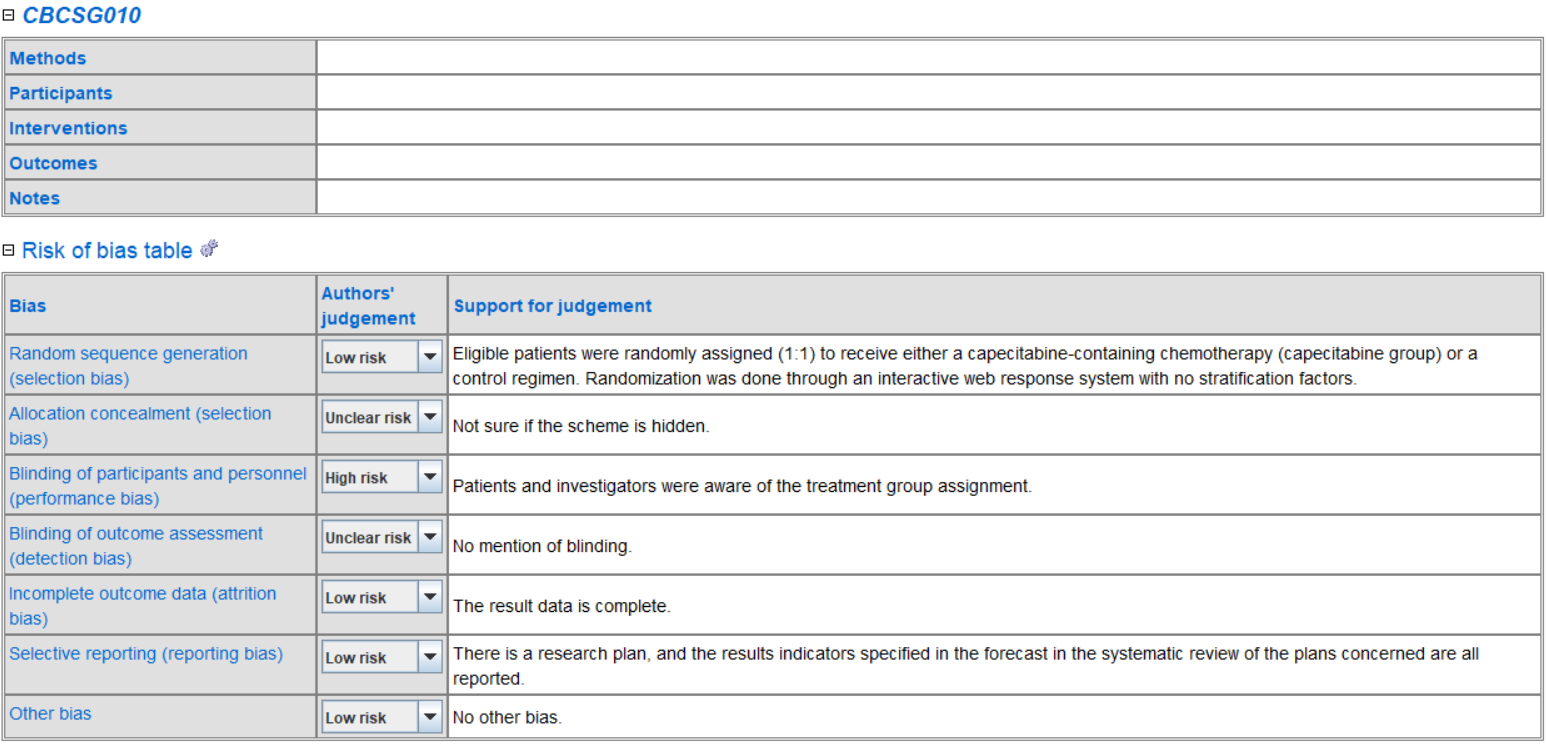


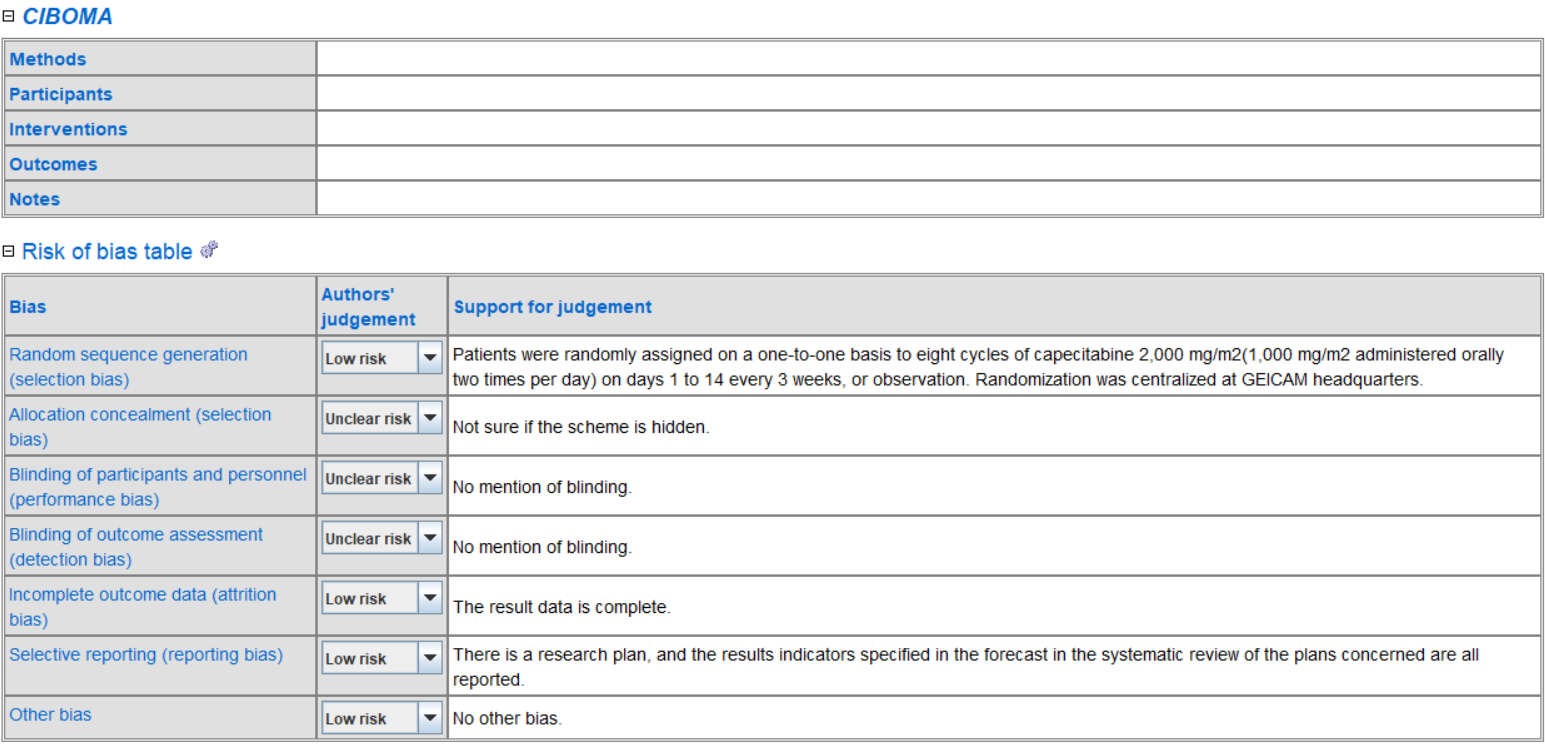


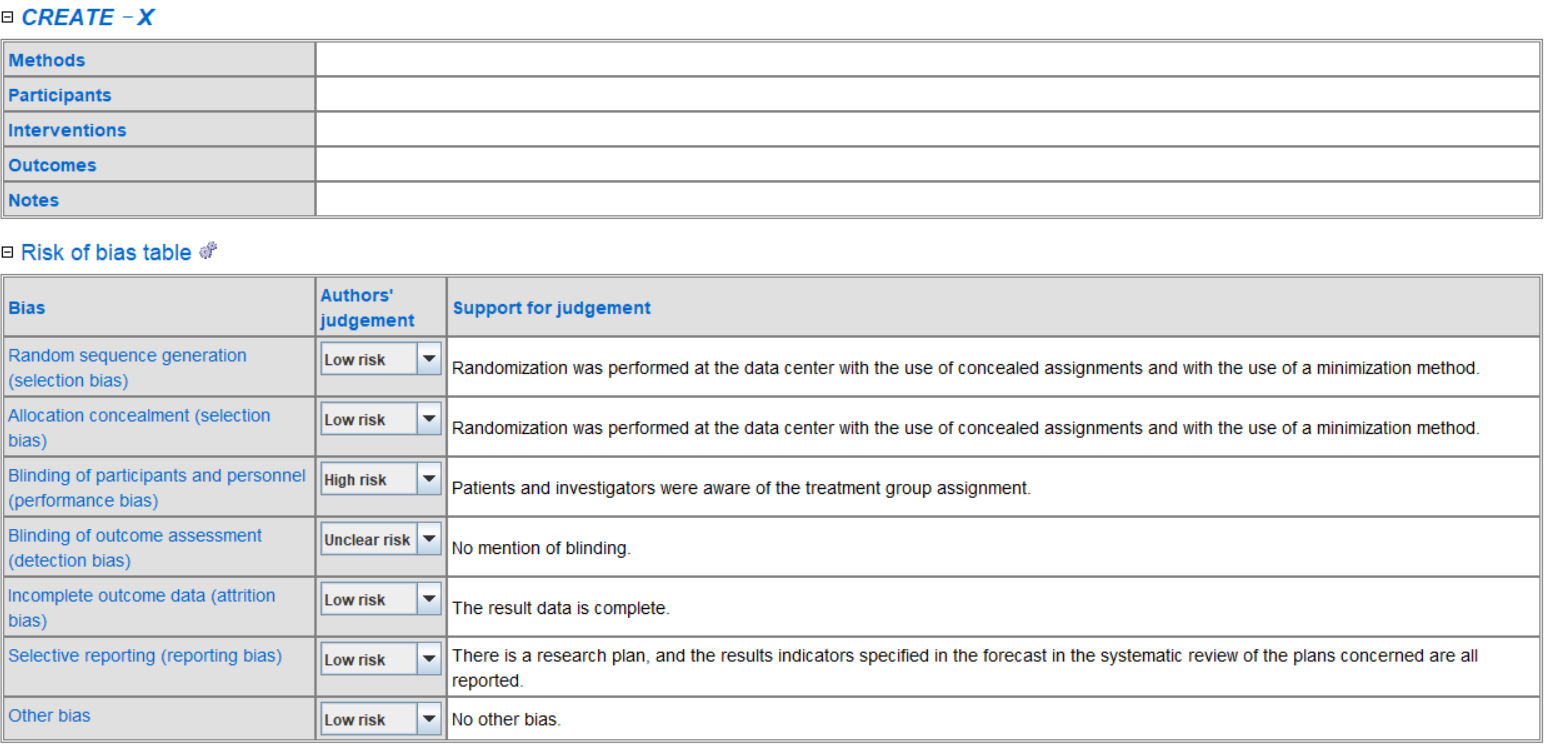


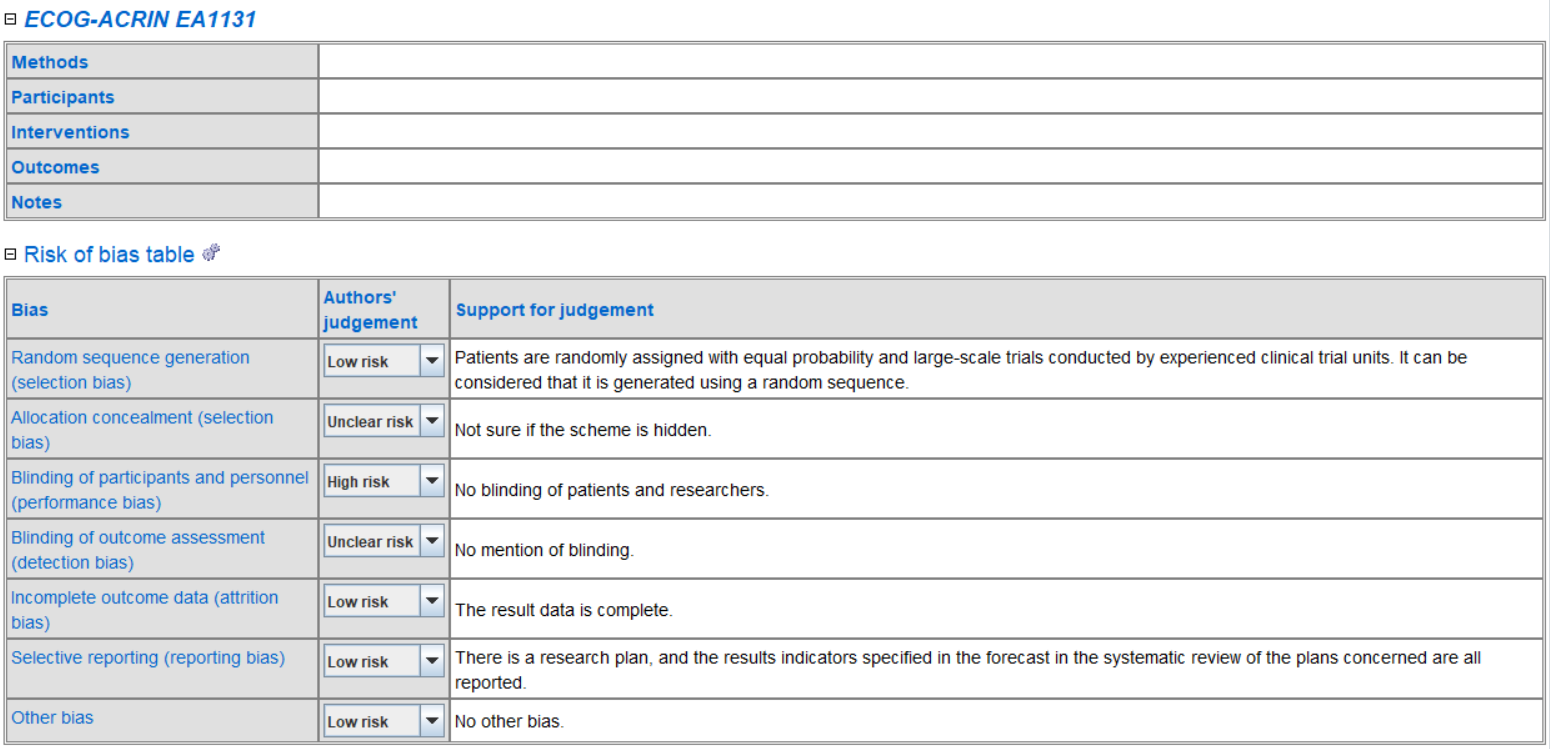


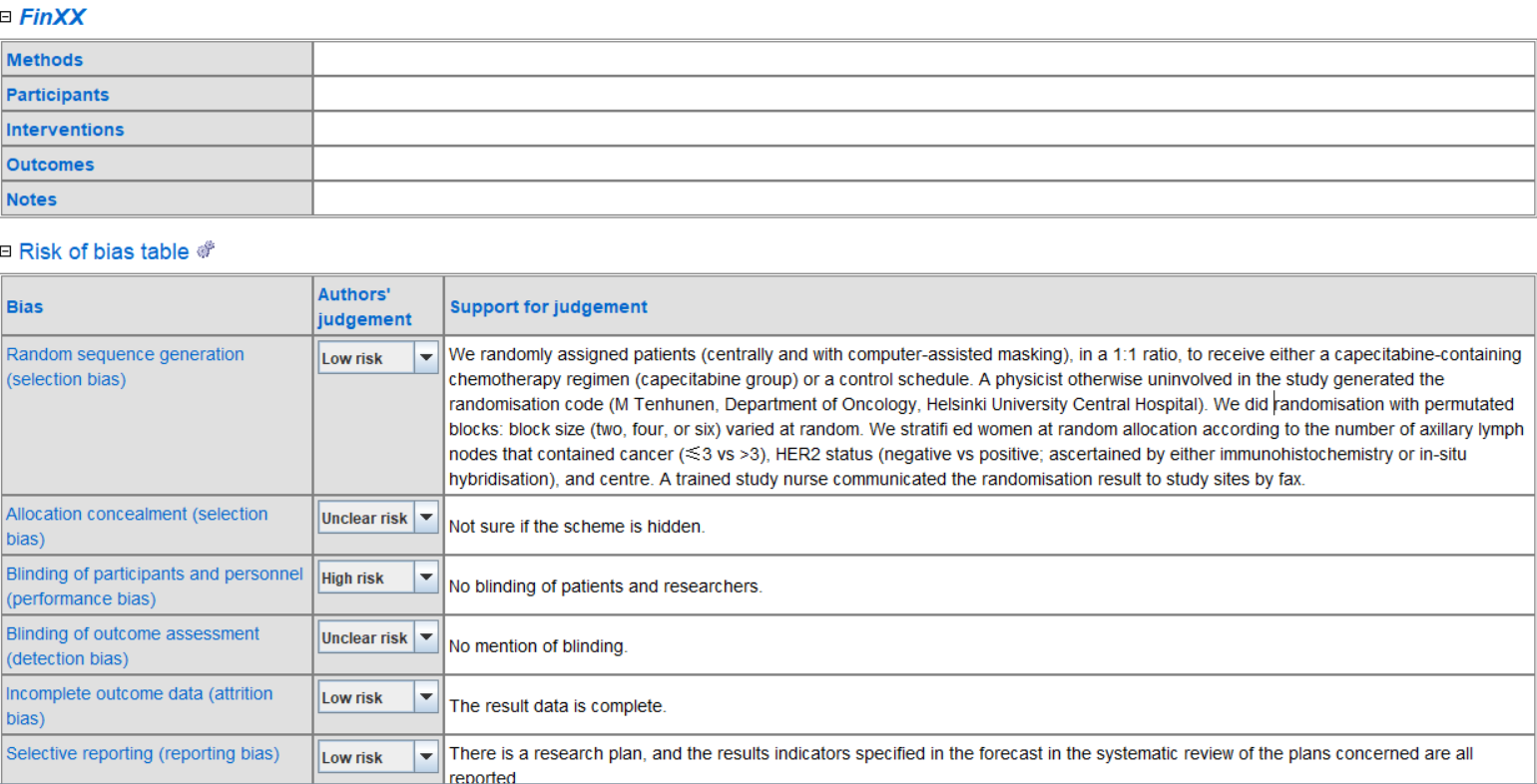


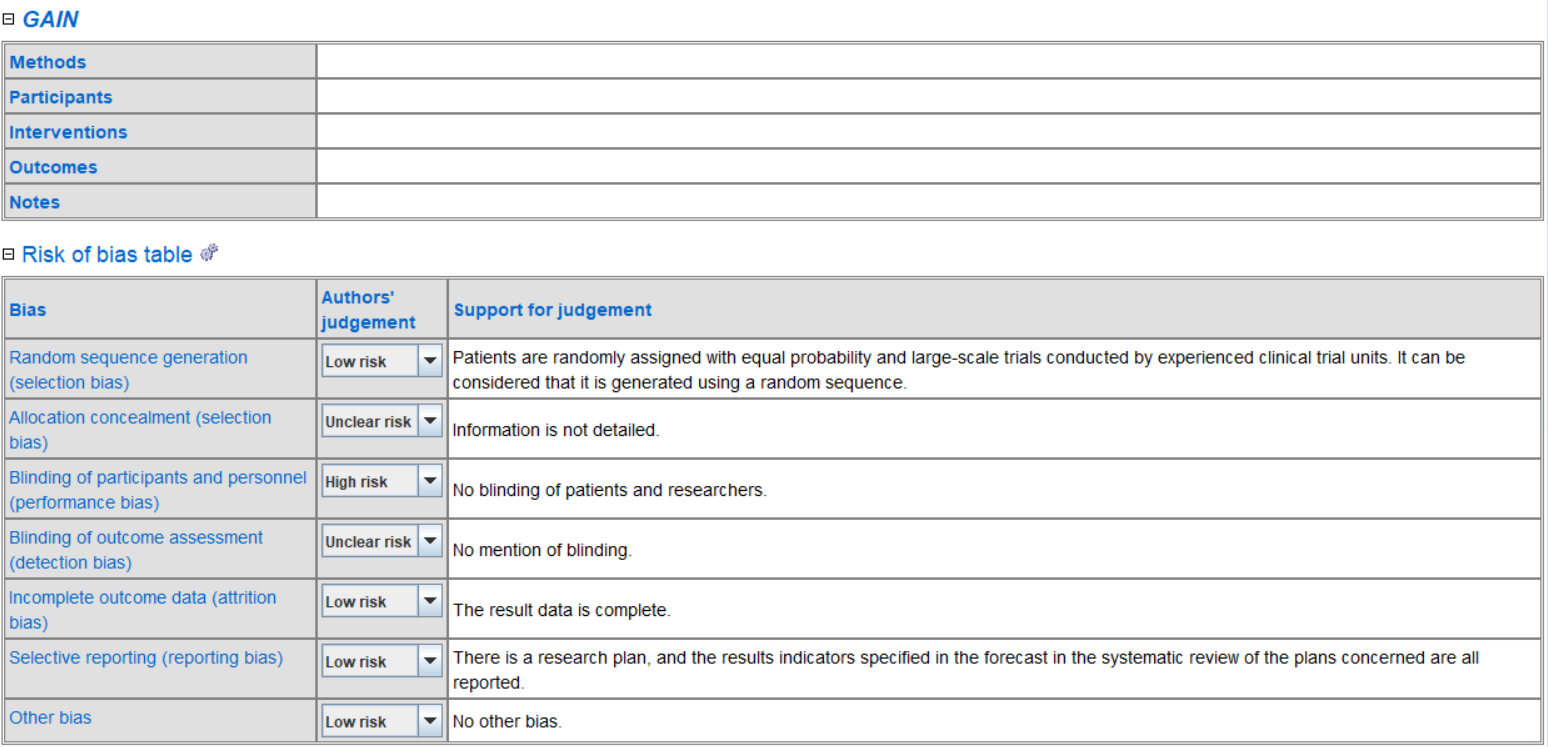


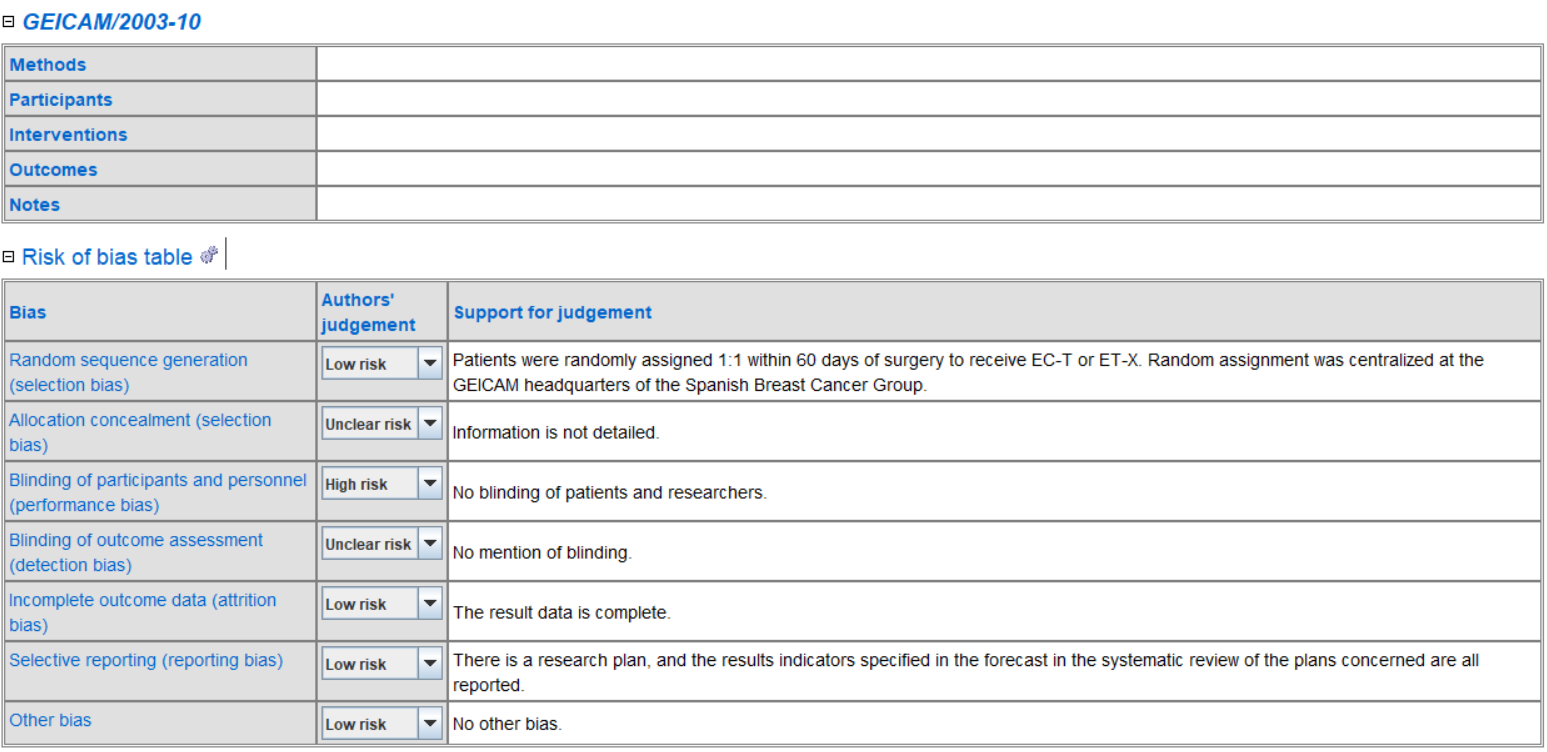

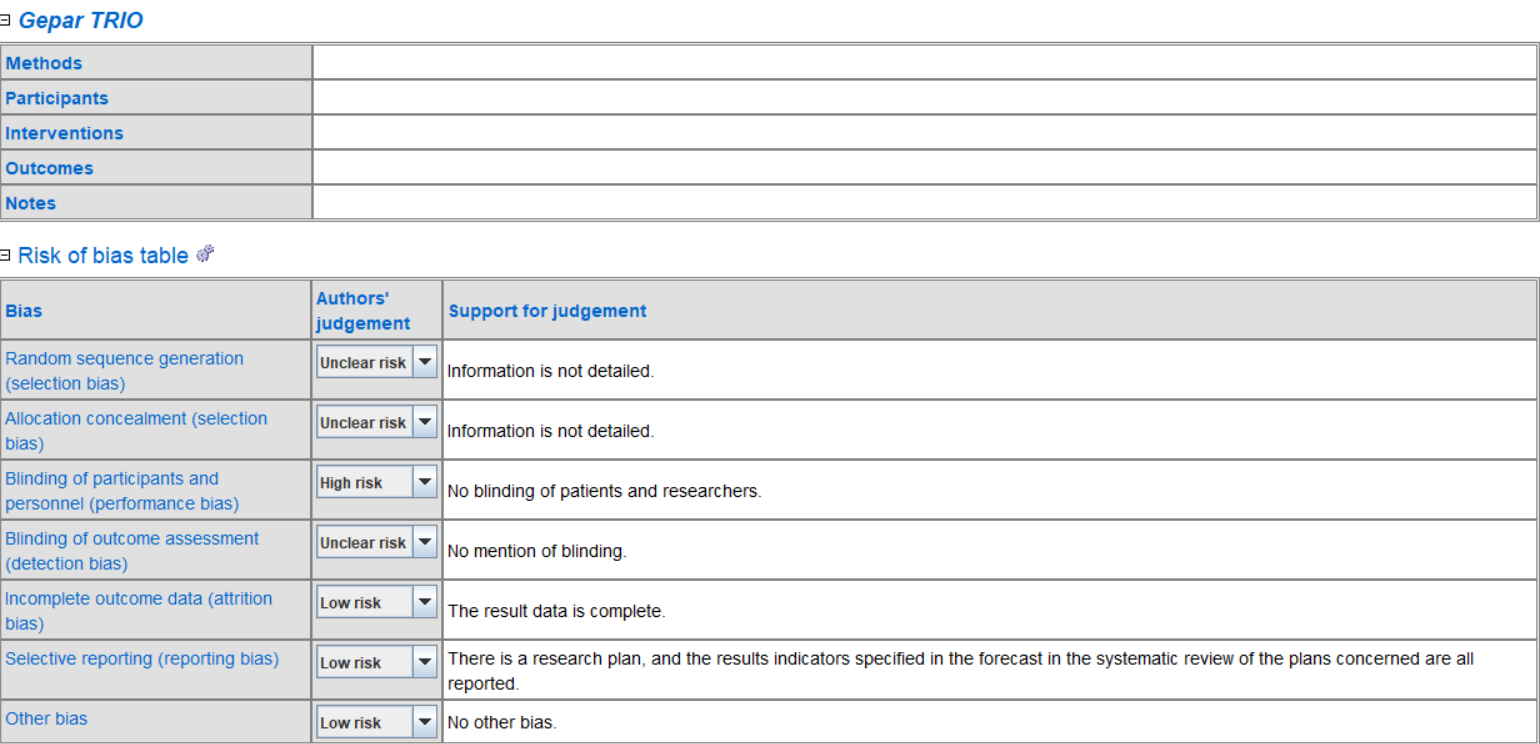


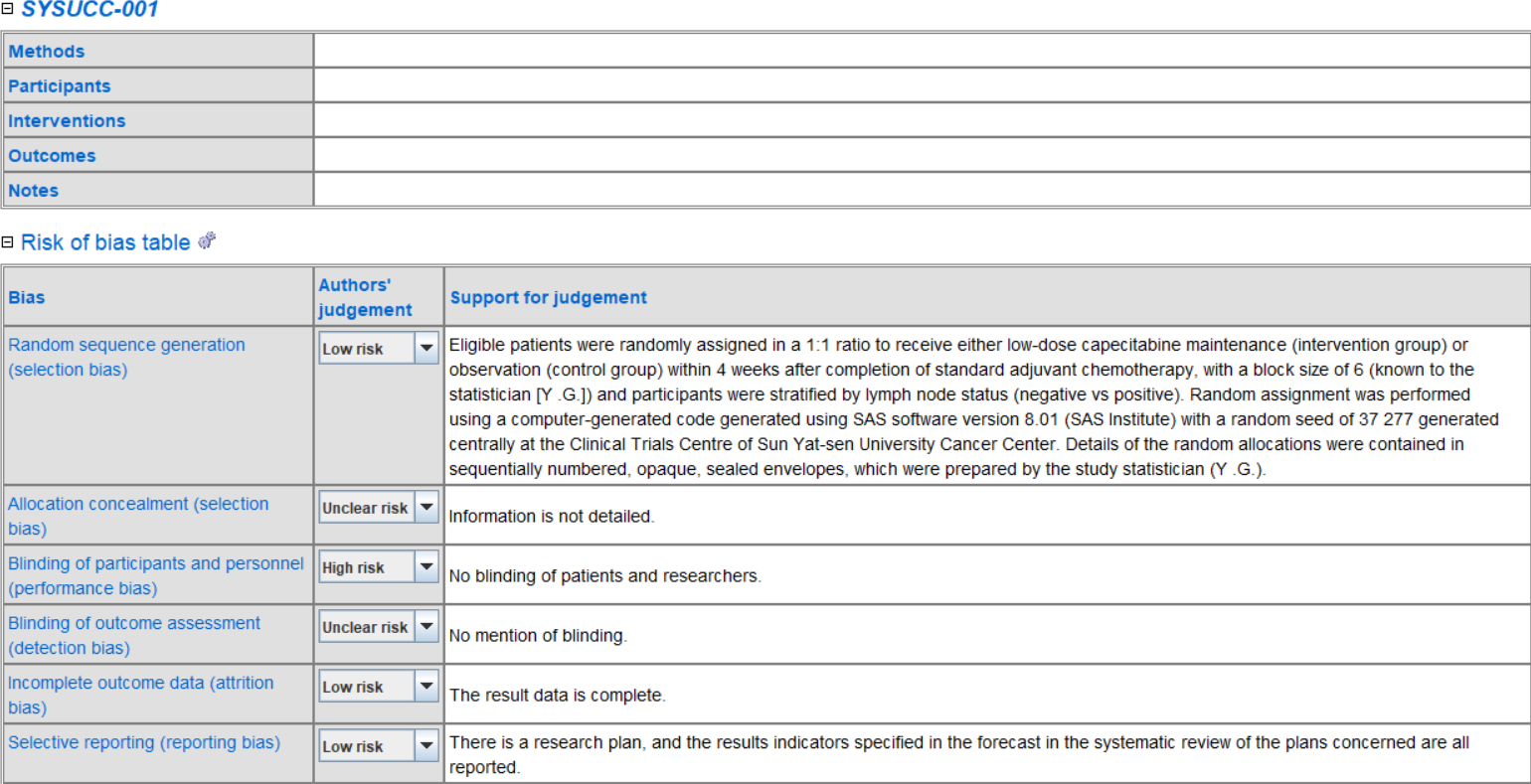


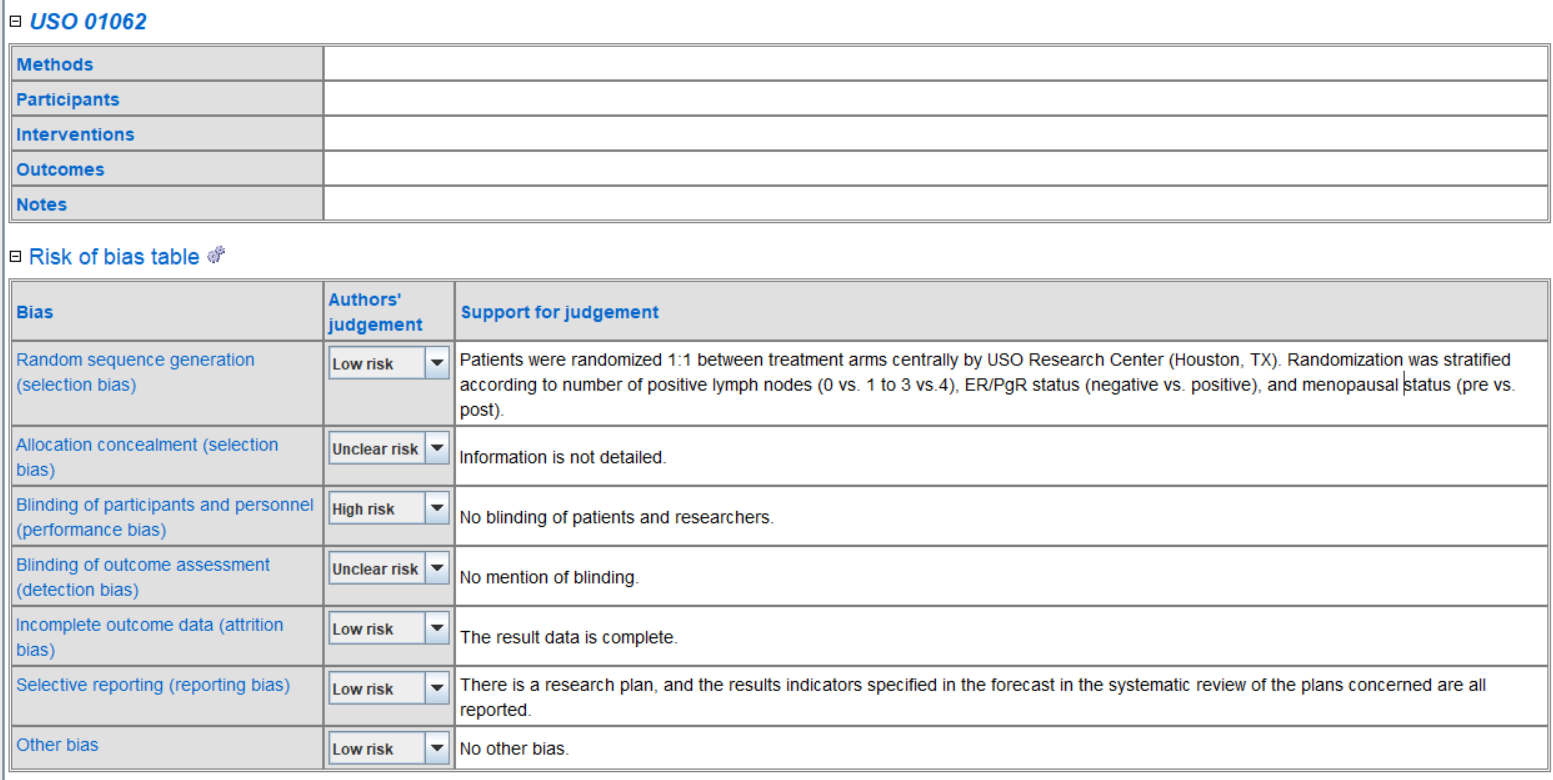


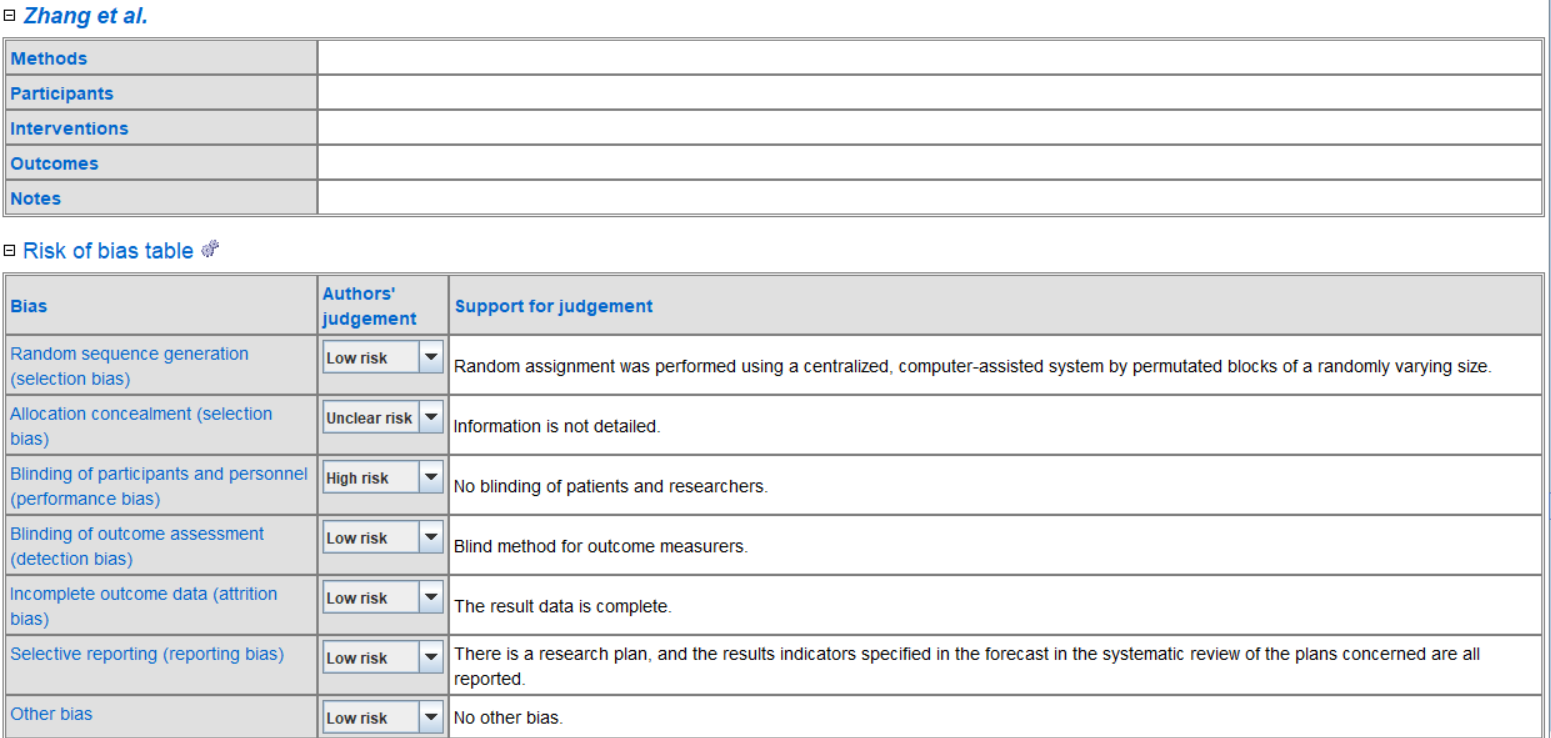


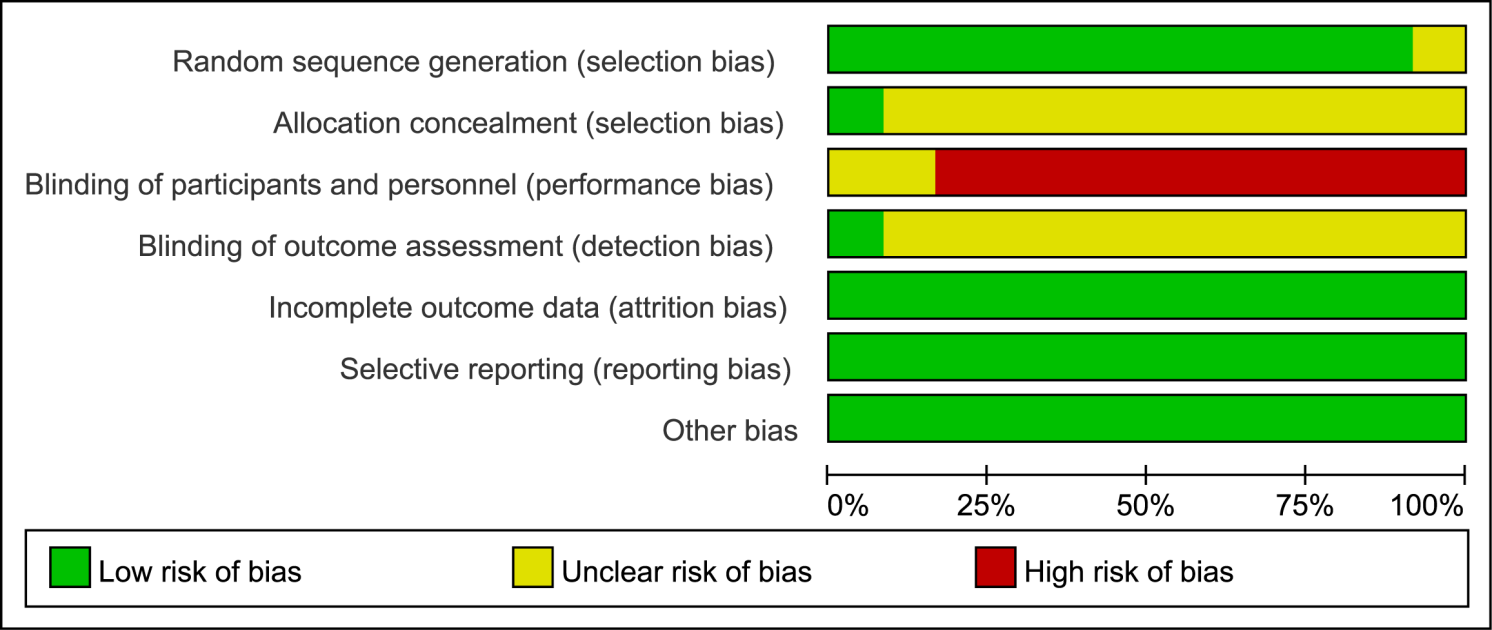


Figure S1．Risk of bias graph


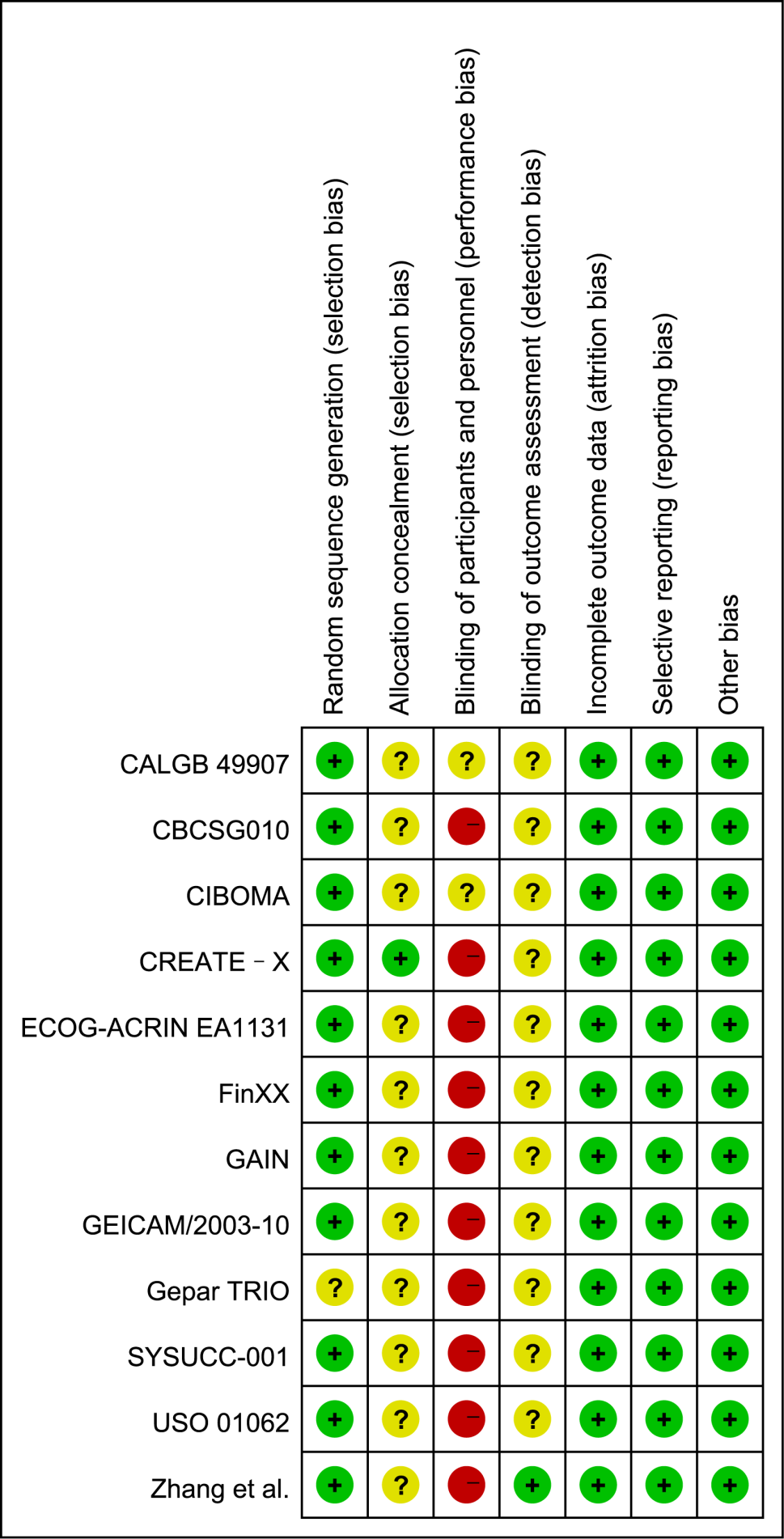


Figure S2．Risk of bias summary
